# Application of a modified tetra-primer ARMS–PCR assay for rapid *Panax* species identity authentication in ginseng products

**DOI:** 10.1038/s41598-023-39940-7

**Published:** 2023-09-01

**Authors:** Zhengxiu Yang, Yat Tung Lo, Zheng Quan, Junchen He, Yanjun Chen, Adam Faller, Tiffany Chua, Hoi Yan Wu, Yanjun Zhang, Qiang Zou, Fan Li, Peter Chang, Gary Swanson, Pang Chui Shaw, Zhengfei Lu

**Affiliations:** 1 Quality Control Laboratory, Herbalife NatSource (Hunan) Natural Products Co., Changsha, 410100 China; 2 Li Dak Sum Yip Yio Chin R & D Centre for Chinese Medicine, State Key Laboratory of Research on Bioactivities and Clinical Applications of Medicinal Plants (CUHK) and School of Life Sciences, The Chinese University of Hong Kong, Shatin, N.T., Hong Kong China; 3 Corporate Center of Excellence Quality Laboratory, Herbalife International of America, Inc., 950 W 190th Street, Torrance, CA 90502 USA; 4 Herbalife International of America, Inc., Corporate Quality, 990 W 190th Street, Torrance, CA 90502 USA

**Keywords:** Genetics, Plant sciences, Biotechnology, Genomics, Molecular engineering, Plant biotechnology

## Abstract

*Panax ginseng* products can be adulterated with materials from other *Panax* species. The purpose of this study is to provide a rapid *P. ginseng* authentication method for simultaneous identification of *P. ginseng* and detection of adulteration in ginseng products at different processing stages. First, a tetra-primer ARMS–PCR assay was designed based on a single-nucleotide polymorphism (SNP) within the *trnL-trnF* region and was tested at 28 PCR cycles with DNA extracted from Botanical Reference Materials (BRMs). Next, 5′ end random nucleotide and 3′ terminus phosphorothioates linkage modifications were incorporated into the inner primers to improve sensitivity and specificity at 40 PCR cycles. Finally, the modified assay was validated using characterized market ginseng materials and the detection limit was determined. The modified tetra-primer ARMS–PCR assay can achieve the desired sensitivity and specificity using one set of reaction conditions in ginseng materials at different stages. In validation, it was able to correctly identify target species *P. ginseng* and differentiate it from closely related species. This study suggests that the modified tetra-primer ARMS–PCR assay can be used for the rapid, species identity authentication of *P. ginseng* material in ginseng products. This assay can be used to complement chemical analytical methods in quality control, so both species identity and processing attributes of ginseng products can be efficiently addressed.

## Introduction

*Panax ginseng*, commonly known as Asian ginseng, has been used in Traditional Chinese Medicine (TCM) for thousands of years. Different from other medicinal plants, *P. ginseng* has multiple closely related species under the genus *Panax*, also in commercial trade. Among them, *P. quinquefolius* (American ginseng) and *P. notoginseng* (Tienchi) are the two other most produced and consumed species^1^. According to a study by Future Market Insights, the global ginseng market size stood at US$ 622.9 Mn in 2019 and is expected to reach US$ 903.8 Mn by the end of 2027^2^. In contrast to growing demand, the prices of ginseng root fluctuate constantly due to supply, the end-user market country, and public perception of each ginseng material. With multiple closely related species co-existing in commercial trade, one major form of adulteration is substituting the claimed ginseng species with another ginseng species. For example, *P. ginseng* may be adulterated with *P. quinquefolius*, or vice versa. Although both *P. ginseng* and *P. quinquefolius* root contain ginsenosides as their bioactive compound and are recognized as adaptogens (herbs to restore equilibrium and resist to adverse factors). In general, they are intended for different therapeutic outcomes.

To prevent adulteration, fit-for-purpose identification method must be used in the quality control of ginseng products. Industry botanical identification often relies on chemical analytical equipment. In many pharmacopeias, chromatographic methods, such as Thin-Layer Chromatography (TLC) and High-Performance Liquid Chromatography (HPLC) methods, have been incorporated for *Panax* species identification based on the presence or absence of characteristic ginsenosides and the ratios between certain ginsenosides^3–5^. However, a sample′s chemical profile is subject to variation, which can be influenced by harvest time, geographic location, storage conditions, and processing. In addition, the possibility of encountering mixed botanical materials in commerce adds another layer of uncertainty when chromatographic methods were developed based on a single botanical material^6,7^. Recently, genomic testing has emerged as an alternative analytical approach to provide species information in botanical quality control. Genomic identification methods utilize characteristic nucleotide sequences, which are specific at the species level and are not subject to change due to environmental factors and the post harvesting process conditions mentioned previously. Sequencing and species-specific PCR are the main approaches to analyze characteristic nucleotide sequences. Compared to sequencing, species-specific PCR requires low capital investment, has a shorter turnaround time, and is more suitable for routine testing. For *P. ginseng* identification, species-specific PCR methods were developed by our groups^8,9^. However, the specificity of PCR amplification depends on the number, type, and location of mismatches between primer and template. Often, it requires searching the entire chloroplast genomes to identity those potential regions with multiple mismatches. In addition, multiple species-specific PCR must be used in combination to detect issues of botanical mixture, which is not uncommon in *P. ginseng* material.

In reality, the differences between close species are sometimes down to a single nucleotide among available DNA sequences. In these scenarios, the amplification refractory mutation system (ARMS)-PCR, also known as allele-specific PCR, becomes an effective choice. Due to the differences in elongation efficiency of Taq DNA polymerase on 3′-end matched and mismatches primers, in ARMS–PCR, species-specific amplification is achieved when the 3′-end of the primers perfectly complement the base at the template^10^. When combined with a tetra-primer system, it can be used to address more than one allele in a single PCR assay, therefore ideal for close species identification and differentiation in mixture form^11^.

Compared to our previous work using regular PCR design strategy for species identification^9^, in this study, we focused on adapting and improving this sophisticated technology and its application in species authentication using well established *Panax spp.* material as an example. In this study, a tetra-primer ARMS-PCR assay was first developed to identify and differentiate *P. ginseng* from other *Panax spp.* and botanicals that have “ginseng” in their common name. Then, multiple modifications on PCR oligos were made to expand its application scope and increase practicality, so that popular industry material types at different processing stages are covered. Finally, the optimized tetra-primer ARMS-PCR assay was validated using authenticated market ginseng root materials at different processing stages, and its performance in detecting adulterants was evaluated by mixing non-target species with *P. ginseng* material, in different ratios.

## Methods

All methods performed and described in this section and supplemental information were following local and international guidelines and legislations. Plant species used in this study were belong to common medicinal plants and commercially available.

### Ginseng reference sequence

The chloroplast genomes of *P. ginseng* (18) (number in parenthesis refers to the number of GenBank accessions evaluated)*, P. quinquefolius* (4), *P. notoginseng* (12)*, P. japonicus* (11)*,* and *Eleutherococcus senticosus* (also known as Siberian ginseng) (1) were obtained from GenBank. All chloroplast genomes were aligned using the MAFFT online service with the default setting^12^ (https://mafft.cbrc.jp/alignment/server/) to obtain a consensus sequence of the same species.

### Reference materials and market samples

BRMs were purchased from American Herbal Pharmacopeia (AHP) (Scotts Valley, CA, US), ChromaDex (Los Angeles, CA, US), Chinese National Institutes for Food and Drug Control (NIFDC) (Beijing, China), US Pharmacopeia (USP) (Rockville, MD, US). The chemical reference standards were purchased from USP and NIFDC. Details about these references are listed in Table 1.

**Table 1:** Reference materials used in current study.

Item	Scientific Name	Supplier	Catalog No	Place used
Asian Ginseng Root	*P. ginseng*	NIFDC	120,917	Figures 1–4, 1S–2S
Asian Ginseng Root	*P. ginseng*	AHP	BRM-PanGinRtWhi	Figures 1–4, 2S
American Ginseng Root	*P. quinquefolius*	AHP	BRM-PanQuiRt	Figures 1–4, Figures 1S–2S
American Ginseng Root	*P. quinquefolius*	ChromaDex	ASB-00030676–005	Figures 1–4, Fig. 2S
Tienchi Ginseng Root	*P. notoginseng*	AHP	BRM-PanPseRt	Figures 1–4, Figures 1S–2S
Tienchi Ginseng Root	*P. notoginseng*	AHP	BRM-PanNotRt	Figures 1–4, 2S
Eleuthero Root	*E. senticosus*	AHP	BRM-ES	Figures 1–4
Asian Ginseng Root	*P. ginseng*	NIFDC	120,917	Figures 5, 3S
Red Asian Ginseng Root	*P. ginseng*	NIFDC	121,045	Figure 4S
American Ginseng Root	*P. quinquefolius*	NIFDC	120,997	Figures 5, 5S
Tienchi Ginseng Root	*P. notoginseng*	NIFDC	120,941	Figure 6S
Powdered Asian Ginseng Extract (1.5 g)	*P. ginseng*	USP	1,291,708	Figure 4
*Panax Notoginseng* Root and Rhizome Dry Extract (1 g)	*P. notoginseng*	USP	1,291,719	Figure 4
Powdered Asian Ginseng Extract (1.5 g)	*P. ginseng*	USP	1,291,708	Figure 3S
Powdered American Ginseng Extract (1.5 g)	*P. quinquefolius*	USP	1,291,683	Figure 5S
*Panax Notoginseng* Root and Rhizome Dry Extract (1 g)	*P. notoginseng*	USP	1,291,719	Figure 6S
Pseudoginsenoside F11	NA	NIFDC	110,841	Figures 3S–6S
Ginsenoside Rc	NA	Bepure	RMT16205	Figures 3S–6S
Ginsenoside Rd	NA	Bepure	RMT16300	Figures 3S–6S
Ginsenoside Re	NA	Bepure	RMT16400	Figure 3S–6S
Ginsenoside Rb1	NA	NIFDC	110,704	Figures 3S–6S
Ginsenoside Rg1	NA	NIFDC	110,703	Figures 3S–6S

All market ginseng products are purchased online (Tables 2 and 2S, Supporting Information).

**Table 2:** Market ginseng products or materials purchased online. BRM: Botanical Reference Material.

Item	Place of origin	Sample attribute	Claimed Species	HPTLC results on species identity
Red Ginseng-1	Jilin, China	Slice	*P. ginseng*	*P. ginseng*
Red Ginseng-2	Jilin, China	Entire root	*P. ginseng*	*P. ginseng*
Red Ginseng-3	Jilin, China	Slice	*P. ginseng*	*P. ginseng*
Red Ginseng-4	Jilin, China	Slice	*P. ginseng*	*P. ginseng*
Red Ginseng-5	Jilin, China	Slice	*P. ginseng*	*P. ginseng*
Red Ginseng-6	Jilin, China	Entire root	*P. ginseng*	*P. ginseng*
American Ginseng-1	Jilin, China	Slice	*P. quinquefolius*	*P. quinquefolius*
American Ginseng-2	Jilin, China	Slice	*P. quinquefolius*	*P. quinquefolius*
American Ginseng-3	Jilin, China	Slice	*P. quinquefolius*	*P. quinquefolius*
American Ginseng-4	Jilin, China	Slice	*P. quinquefolius*	*P. quinquefolius*
American Ginseng-5	Jilin, China	Slice	*P. quinquefolius*	*P. quinquefolius*
American Ginseng-6	Jilin, China	Slice	*P. quinquefolius*	*P. quinquefolius*
Tienchi-1	Yunnan, China	Dry root	*P. notoginseng*	*P. notoginseng*
Tienchi-2	Yunnan, China	Dry root	*P. notoginseng*	*P. notoginseng*
Tienchi-3	Yunnan, China	Dry root	*P. notoginseng*	*P. notoginseng*
Japanese Ginseng-1	Anhui, China	Dry root	*P. japonicus*	Not evaluated
Japanese Ginseng-2	Anhui, China	Dry root	*P. japonicus*	Not evaluated
Japanese Ginseng-3*	Anhui, China	Dry root	*P. japonicus*	Not evaluated
Acanthopanax Root-1	Heilongjiang, China	Slice	*E. senticosus*	Not evaluated
Acanthopanax Root-2	Heilongjiang, China	Slice	*E. senticosus*	Not evaluated
Acanthopanax Root-3	Heilongjiang, China	Slice	*E. senticosus*	Not evaluated

*Not used in validation due to HPTLC profile difference between lot No. 3 and the other two lots. See Supplementary Fig. 5 for details.

### Decoction preparation

The decoctions were prepared as follows. For each sample, 5 g of root slices was added to 100 mL of distilled water, heated on a heating plate, and boiled for 30 min. During the boiling stage, distilled water was added to maintain the total volume of 100 mL. The mixture was left to cool down to room temperature and the supernatant was centrifuged at 12,100×*g* to remove solid tissues, then frozen and lyophilized.

### DNA extraction

All ginseng root materials were ground into fine powders using the MiniG 1600 (SPEX SamplePrep, Metuchen, NJ, USA) before extraction. For ginseng root powders, genomic DNA was extracted using the DNeasy mericon Food Kit (QIAGEN, Hilden, Germany) with input powder weight around 50 mg. The extracted genomic DNA was eluted into 150 μL of the elution buffer, then quantified using a Qubit 3.0 Fluorometer (ThermoFisher Scientific, Waltham, MA, USA). For ginseng extracts, the same standard protocol was used with input powder weight increased to 400 mg and the elution volume decreased to 50 μL.

### Tetra-primer ARMS-PCR condition

**Figure 1:**
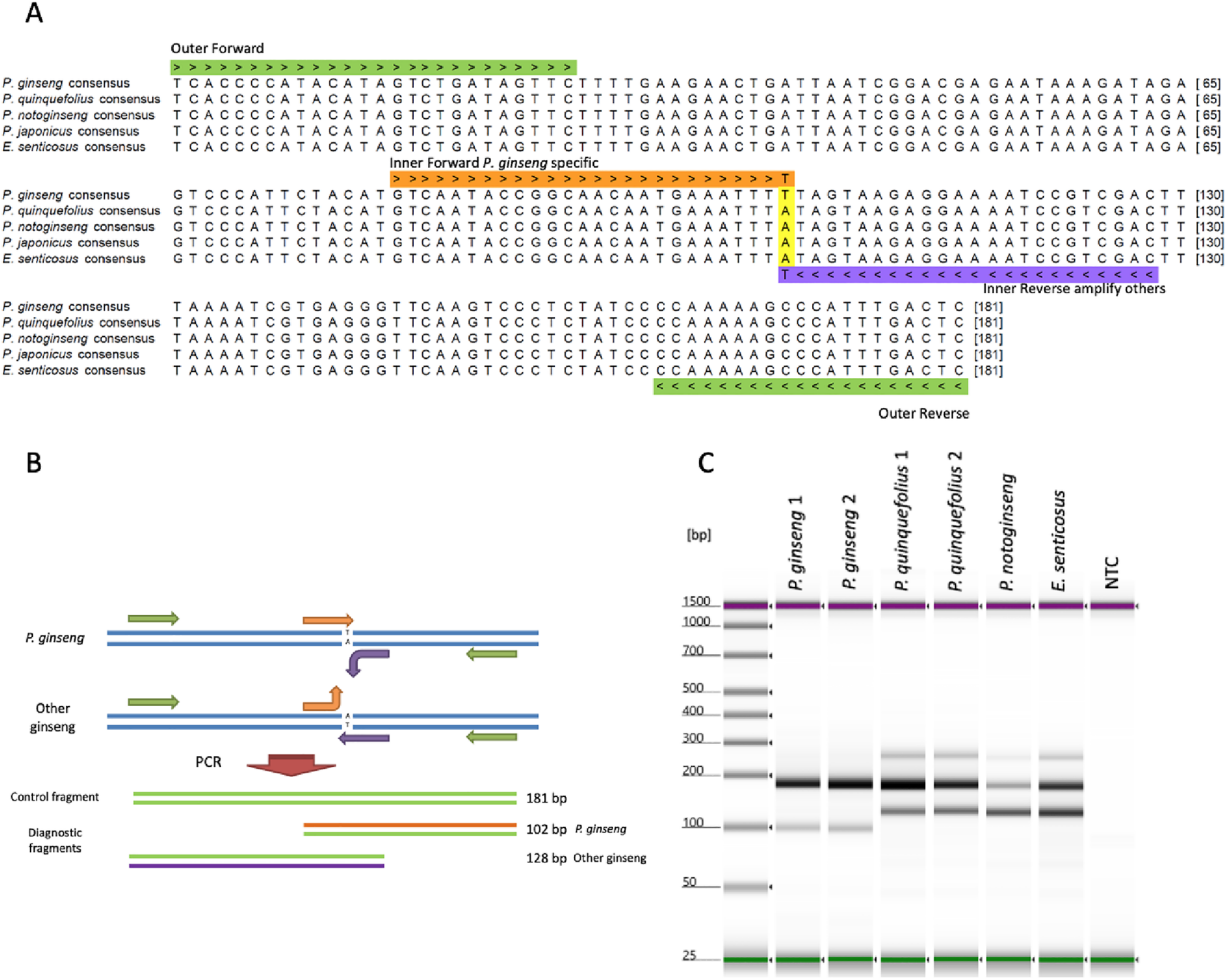
Design and application of tetra-primer ARMS-PCR for *P. ginseng* root identification. (**A**) Tetra-primer location and alignment of partial *trnL-trnF* intergenic region sequences*.* (**B**) A schematic diagram of amplification products. (**C**) Amplification results from BRMs DNA after 28 cycles. NTC: non-template control.

**Figure 2:**
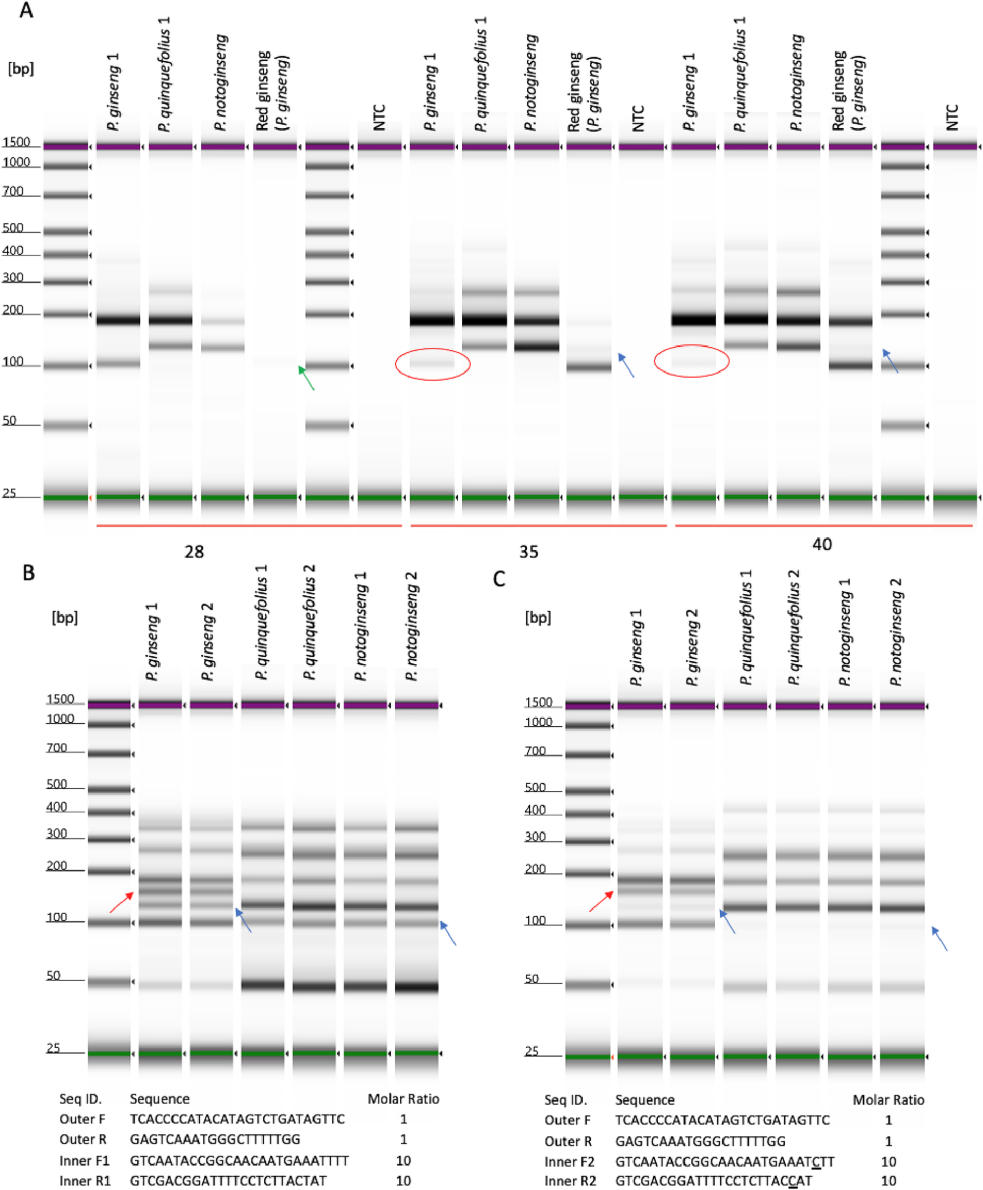
Visualization of amplification results from BRMs and red ginseng DNA used to design tetra-primer ARMS-PCR. (**A**) Amplification results at 28, 35, and 40 PCR cycles. (**B**) Amplification results with ten-to-one inner *vs.* outer primer ratio at 40 cycles. (**C**) Amplification results with ten-to-one inner versus outer primer ratio and deliberate mismatch at − 2 position at 40 cycles. Green arrow: faint PCR product at 28 cycles. Red circles: decreased band intensity at 35 and 40 cycles versus 28 cycles. Blue arrows: non-specific amplification of diagnostic bands. Red arrows: non-specific amplification of unexpected bands.

**Figure 3:**
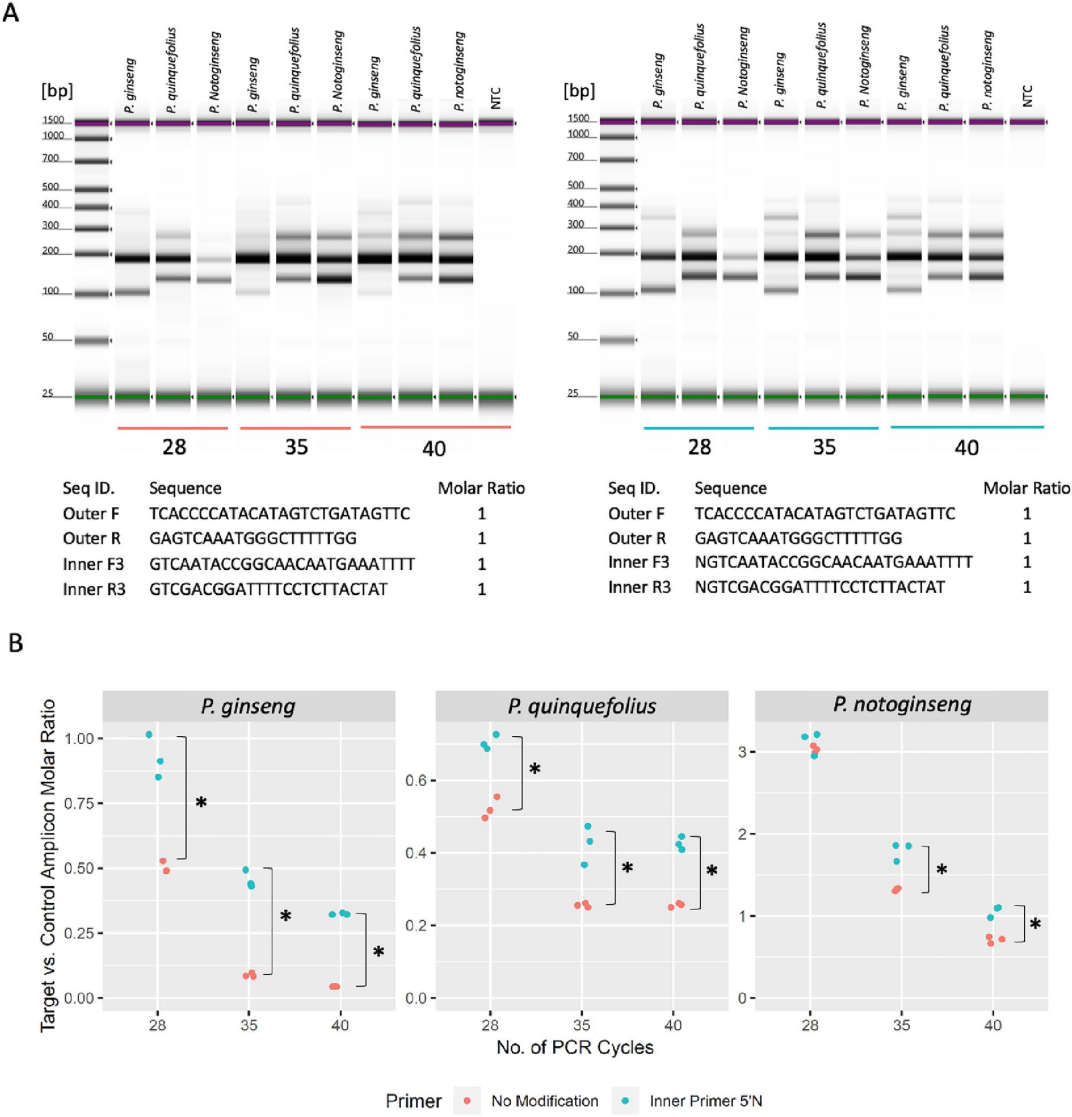
Adding 5′ terminus random nucleotides increases the intensity of diagnostic bands. (**A**) Amplification results using equal ratio of inner and outer primers at various PCR cycles. Left: regular inner primer; right: inner primer with 5′ terminus random nucleotides. (**B**) Target versus control band molar ratio statistical analysis using regular inner primers (red) and 5′ terminus modified inner primers (Cyan).

**Figure 4:**
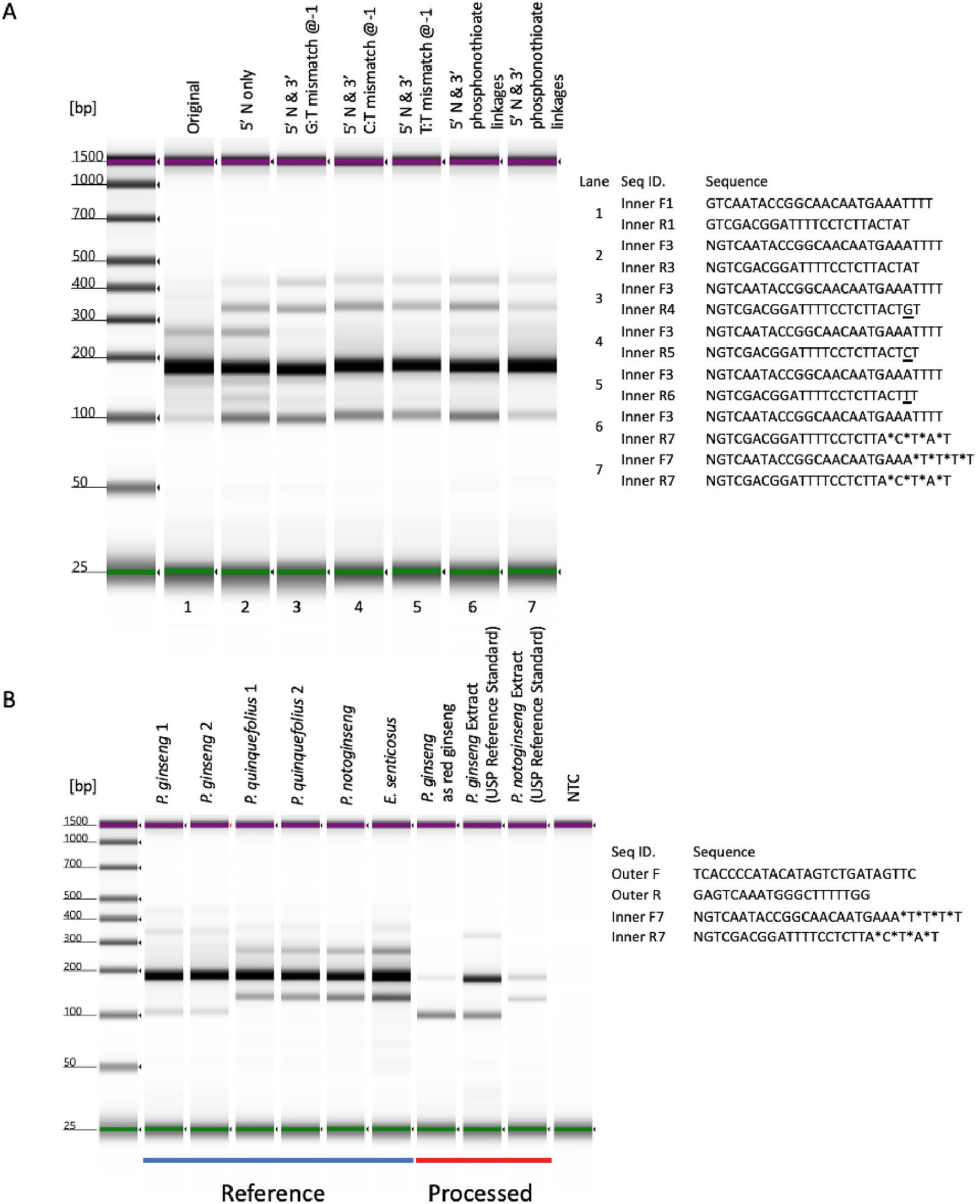
Adding 3′ terminus nucleotide modification increases the specificity of the assay at high PCR cycles. (**A**) Amplification results using inner primer with different 3′ terminus nucleotide modifications at 40 PCR cycles. (**B**) Amplification results from BRMs and processed materials using inner primer with both 5′ terminus and 3′ terminus modifications at 40 PCR cycles.

All primers were synthesized by Integrated DNA Technologies (IDT, Coralville, IA, USA) with standard desalting. Outer forward primer: 5′-TCACCCCATACATAGTCTGATAGTTC-3′, outer reverse primer: 5′- GAGTCAAATGGGCTTTTTGG-3′, regular inner forward primer: 5′- GTCGACGGATTTTCCTCTTACTAT-3′, regular inner reverse primer: 5′- GTCAATACCGGCAACAATGAAATTTT-3′, modified inner forward primer: 5′- NGTCGACGGATTTTCCTCTTA*C*T*A*T-3′, modified inner reverse primer: 5′- NGTCAATACCGGCAACAATGAAA*T*T*T*T-3′. (Note: N, random nucleotides; *, phosphorothioate bond). Other forms of inner primers were also synthesized as indicated in Figs. 2–4 and Figs. 1S–2S (Supporting Information). The tetra-primer ARMS-PCR assay was performed in a 20 μL PCR mix, containing 10 μL AmpliTaq Gold™ 360 Master Mix, 2 μL primer mix (with 5 μM each primer), 2 μL DNA extracted from ginseng materials, and 6 μL nuclease-free water. The following thermal profile was used: (1) an initial step of 3 min at 95 °C and (2) various cycles (28–40) of 30 s at 95 °C, 30 s at 60 °C, and 30 s at 72 °C and (3) final extension 3 min at 72 °C. For gDNA extracted from ginseng extract, 6 μL was used. A positive control, DNA isolation control, and negative control were included in each run and products were visualized either by TapeStation 4200 with D1000 tape (Agilent Technologies, Santa Clara, CA), LabChip (PerkinElmer, Waltham, MA, US), or 2% agarose gel with GelRed (Biotium, Fremont, CA, US).

PCR condition optimization was performed in the Herbalife Center of Excellence (COE) Laboratory. Validation was performed in the Herbalife NatSource Quality Control Laboratory and laboratory at The Chinese University of Hong Kong.

### Statistical analysis

Welch′s t-test (two-sided) was performed in R software and figures were produced using the package ggplot2 to analyze the changes between inner small and outer large fragment molar ratio^13,14^. *P*-values < 0.05 were considered to indicate statistical significance.

## Results

### Design and application of tetra-primer ARMS-PCR for *P. ginseng* root identification

The *trnL-trnF* intergenic region, which contains a *P. ginseng* specific SNP, was selected for designing the tetra-primer ARMS-PCR assay due to its balanced GC content. As shown in Fig. 1A, four primers were designed around the yellow highlighted SNP position to give PCR fragments in different lengths for *P. ginseng* (102 bp), other ginseng species (128 bp), and control (181 bp) (Fig. 1B). The current tetra-primer ARMS-PCR was applied to genomic DNA extracted from reference materials of *Panax spp.* and *E. senticosus* roots. As shown in Fig. 1C, after 28 cycles of PCR amplification, DNA extracted from *P. ginseng* reference materials generated a large fragment around 180 bp and a small fragment around 100 bp, while DNA from *P. quinquefolius*, *P. notoginseng*, and *E. senticosus* yielded the same 180 bp large fragment and another distinctive small fragment with size around 130 bp. Therefore, based on the size difference between small fragments, *P. ginseng* root could be clearly distinguished from roots of other species in the current test scope.

### Evaluate applicability of current assay for the identification of *P. ginseng* products

Due to DNA degradation caused by manufacturing processes, performing PCR with highly processed ginseng materials is challenging. Figure 2A shows the amplification profiles generated using DNA extracted from ginseng BRMs along with previously authenticated red ginseng root (steamed root, representing highly processed product) at various PCR cycles. For DNA from regular ginseng roots, the condition with 28 PCR amplification cycles worked as expected. However, in red ginseng, the same condition only yielded a faint small inner fragment (green arrow). With the increase of PCR cycle number, the once faint or undetectable fragments gained intensity, suggesting the current assay was able to generate a characteristic amplification profile from degraded DNA in highly processed *P. ginseng* products. However, under the same high PCR cycle number condition, the other 130 bp inner band, which indicated the presence of other ginseng species, also became noticeable (blue arrows). Since the red ginseng DNA was extracted from a single steamed root, the presence of both 102 bp and 128 bp diagnostic bands indicated a decrease of specificity for the current tetra-primer ARMS-PCR when used at high cycle number required for processed material. In contrast, for DNA from regular ginseng roots, high PCR cycle numbers led to lower sensitivity due to the intensity reduction in the small diagnostic fragment, especially for the target species *P. ginseng* (red circles).

### Classical strategy to improve *P. ginseng* tetra-primer ARMS-PCR sensitivity and specificity

To improve both the sensitivity and specificity of the current assay, ten-to-one inner *vs.* outer primer ratio and additional deliberate mismatch at − 2 position from 3′ terminus of inner primers were tested according to a tetra-primer ARMS-PCR design website (http://primer1.soton.ac.uk/primer1.html). As demonstrated in Fig. 2B, with 40 cycles of PCR amplification, simply increasing the inner *vs.* outer primer ratio yielded a well-balanced small and large fragment intensity ratio. However, the specific amplification of inner fragment was also lost (blue arrows, Fig. 2B). Adding deliberate mismatch at − 2 position did reverse the loss of specificity (Fig. 2C). However, the amplification by other diagnostic fragments was not eliminated (blue arrows, Fig. 2C). Furthermore, non-specific amplifications that may complicate data interpretation were more frequently observed in *P. ginseng* samples (red arrows, Fig. 2B,C). Current observation suggests that the classical strategy does not achieve the desired sensitivity and specificity. An alternative solution is required.

### Increase diagnostic bands intensity by 5′ terminus random nucleotides

In the classical strategy, excessive number of inner primers were supplied to compensate inner primer degradation caused by the 5′ to 3′ exonuclease activity of Taq DNA polymerase. However, previous attempts showed that it also resulted in non-specific amplification of the inner fragment of the other allelic region, which would be mistaken as the presence of adulterates. Introducing a nucleotide mismatch in the inner primer would create certain levels of hybridization preference between the inner primer and newly synthesized small fragments; thus, reducing the level of inner primer degradation caused by Taq DNA polymerase. To test this hypothesis, an inner primer set with a 5′ terminus random nucleotide was compared to a regular inner primer. Figure 3A demonstrates that replacing the regular inner primer with an inner primer set with a 5′ terminus random nucleotide boosted the intensity of inner fragment across various PCR cycles (right *vs.* left panel). To further quantify the improvement of assay sensitivity across different PCR cycles, the molar ratio between the desired inner fragment and control fragment was obtained in triplicate (Fig. 3B). At 35 and 40 cycles, higher inner small *vs.* outer large fragment molar ratio was achieved when modified inner primers were used (*p* < 0.05, Welch′s *t*-test), suggesting adding 5′ terminus random nucleotides on inner primers indeed improves the assay sensitivity. In addition to random nucleotides on the 5′ terminus, additional variations of mismatches at the 5′ terminus and in the middle region were also tested (Fig. 1S).

### Maintain assay specificity at high PCR cycles by 3′ terminus nucleotide modification

In the previous study, two mismatches within each inner primer (one at the end of 3′ terminus and one at − 2 position from the 3′ terminus) were not sufficient to inhibit the non-specific amplification of the diagnostic bands at 40 PCR cycles (Fig. 2C). To further improve the specificity of the assay, two additional types of modifications were tested: (1) introducing deliberate mismatch at penultimate base (− 1) instead of − 2 position to deliver a more direct effect on decreasing elongation efficiency^15^; (2) replacing the 3′ end regular phosphodiester inter-nucleotide linkages with phosphorothioate linkages. As shown in Fig. 4A, while mismatch at the penultimate base did further reduce the amplification of the non-specific inner fragment, incorporating four consecutive phosphorothioate linkages at 3′ terminus completely blocked non-specific amplification. Apply primers with 3′ phosphorothioate linkages to the same set of BRMs used in assessing classical strategy confirmed the improved specificity (Fig. 4S). To achieve desired sensitivity and specificity, both 5′ end random nucleotide and 3′ terminus phosphorothioate linkage modifications were incorporated into the inner primers and used as a modified tetra-primer ARMS-PCR. Our result indicated that, with the modified inner primers, a universal *P. ginseng* identification assay condition could be achieved with acceptable sensitivity and specificity (Fig. 4B).

### Validation of tetra-primer ARMS-PCR using commercial ginseng products

**Figure 5:**
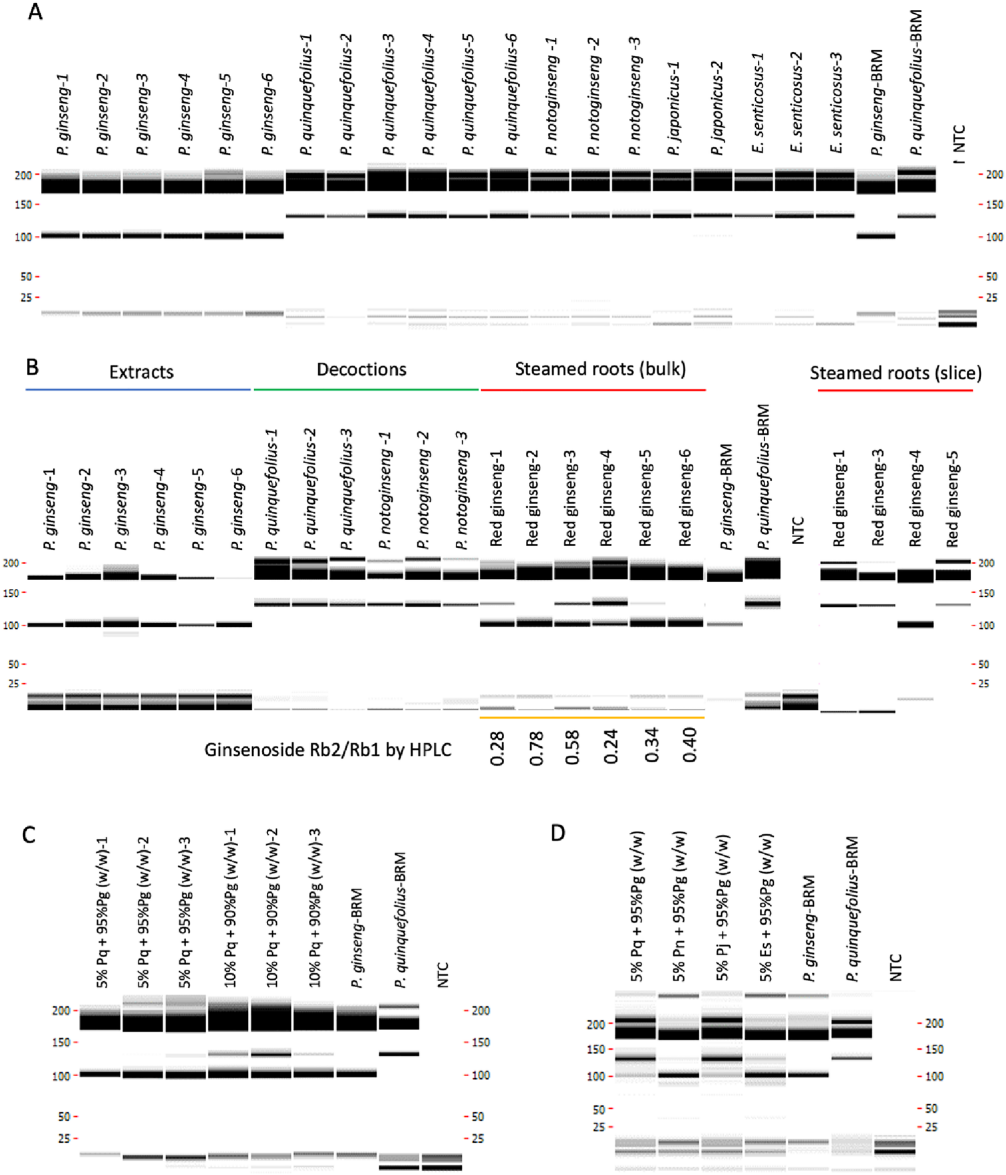
Authentication of marketed ginseng materials using modified tetra-primer ARMS-PCR assay. (**A**) Amplification results of market samples at 40 PCR cycles. (**B**) Amplification results of extracts*,* decoctions*,* red ginseng bulk material, red ginseng individual slice and BRMs at 40 PCR cycles. The texts below the orange bar list ginsenoside Rb2/Rb1 ratio obtained from red ginseng bulk using UHPLC method. (**C**) Amplification results of *P. ginseng* root mixed with various weight percentage of *P. quinquefolius* root. Pq*: P. quinquefolius;* Pg*: P. ginseng.* (**D**) Amplification results of decoctions made from *P. ginseng* root mixed with 5% (w/w) of roots from other ginseng species root. Pn: *P. notoginseng;* Pj: *P. japonicus;* Es*: E. senticosus*.

**Figure 6:**
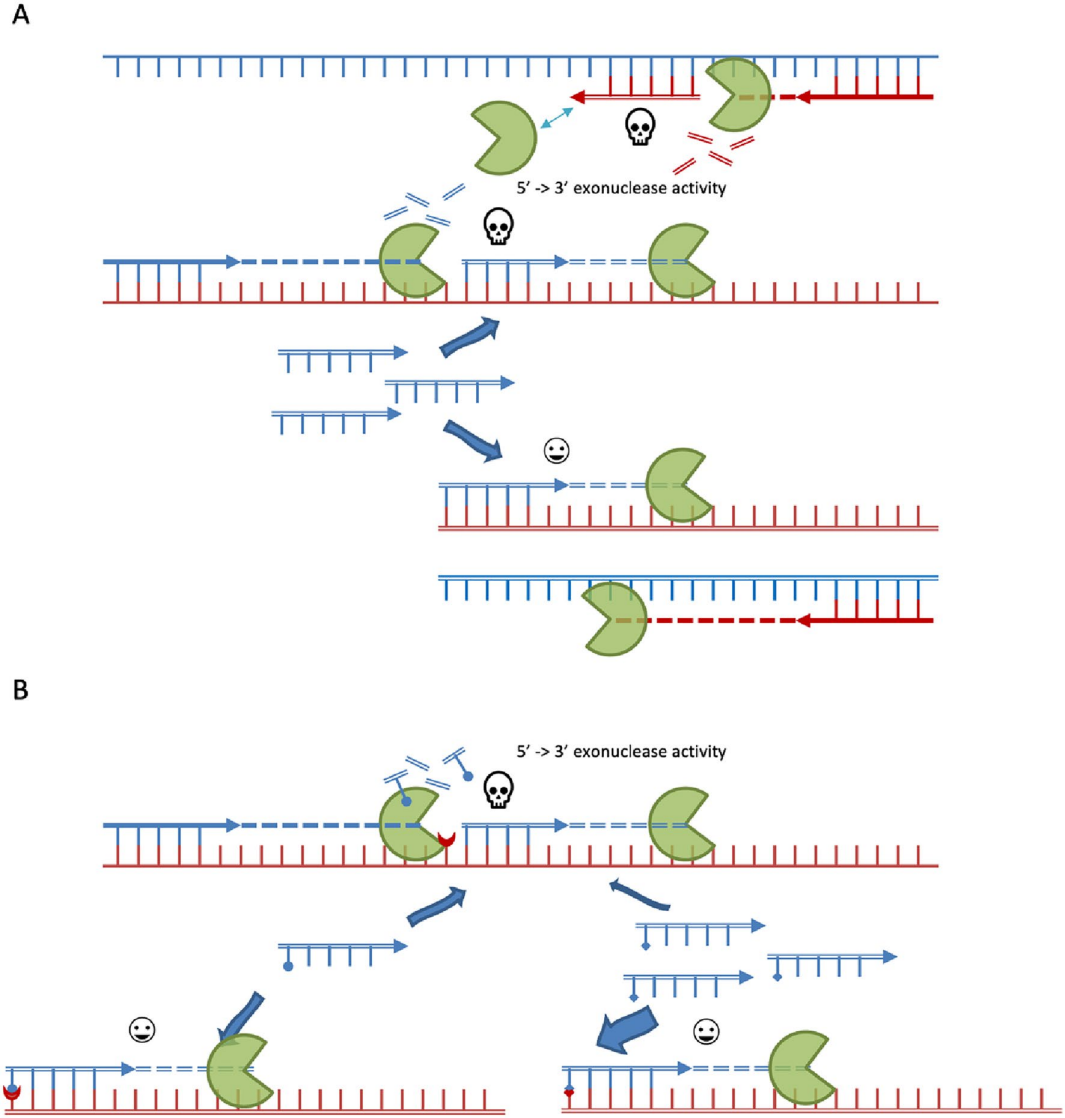
Schematic mechanism diagram to illustrate sensitivity improvement in current modified tetra-primer ARMS-PCR assay by replacing original inner primers with 5′ end random nucleotide inner primers. Pac-man shaped circular sector: Taq DNA polymerase; Line with ticks: top (blue) and bottom (red) DNA strand; Arrow line with ticks: outer forward (blue) and reverse (red) primers (solid line) and inner forward (blue) and reverse (red) primers (double line); skull symbol: primer or newly synthesized DNA strand will be degraded by Taq DNA polymerase; happy face symbol: primer or newly synthesize DNA strand unaffected. Circles and squares on tick represent random nucleotides that match or do not match DNA template. The size of the block arrows indicates the distribution of inner primers annealed to DNA template. (**A**) Regular inner primers bind to long and short DNA template at same affinity. (**B**) Inner primers with 5′ end terminal random nucleotides bind to newly synthesized short DNA template contains random sequence induced mutations with a better affinity than long DNA template with wild-type sequence. Only forward inner primer binding scenario is shown.

To validate the modified tetra-primer ARMS-PCR for testing materials at different processing stages, market *P. ginseng* samples were collected from online, characterized by compendial or proposed compendial analytical methods (Figs. 3S–6S and Chemical analysis, Supporting Information) and used to assess the performance of the current method. For unprocessed or lightly processed root samples (drying only), the current assay was able to identify six lots of *P. ginseng* samples as *P. ginseng*, and assign multiple lots of *P. quinquefolius*, *P. notoginseng*, *P. japonicus*, and *E. senticosus* to other ginseng species based on their distinctive PCR patterns (Fig. 5A). In addition, two types of highly processed ginseng samples were also used. For ginseng extracts, the current assay was able to identify *P. ginseng* extract samples (manufactured at industry scale, blue section in Fig. 5B) as *P. ginseng*, and give different patterns for *P. quinquefolius* and *P. notoginsneg* decoction, made from previously authenticated root samples (green section in Fig. 5B). However, for steamed root, testing on red ginseng samples yielded inconsistent amplification profiles (red sections in Fig. 5B). Further examination of the samples revealed they exist in different forms (Fig. 7S, Supporting Information). Two samples yielded *P. ginseng* patterns were in the form of whole root (red ginseng-2 and 6), while the other four samples in the form of root slices gave patterns that suggesting a mixture of *P. ginseng* and other ginseng species. To investigate the possibility that red ginseng root slices contain material from different botanical origins, additional DNA extraction were performed using individual slice. As shown in Fig. 5B [steamed roots (slice) portion], the amplification profile indicated the tested slice was either *P. ginseng* or belonged to another species.

To further characterize the chemical profile of these red ginseng samples, additional UHPLC analysis was performed and ginsenoside Rb2/Rb1 ratios (which is a key indicator for ginseng differentiation) were measured (orange section in Fig. 5B and Table 1S, Supporting Information). Results of the Rb2/Rb1 ratio from three out of four lots of red ginseng samples that tested positive for containing mixed species ranged from 0.22 to 0.35, while the Rb2/Rb1 ratio for the red ginseng lots that yield pure *P. ginseng* profile were 0.78 and 0.37. However, all the samples passed the Rb2/Rb1 ratio acceptance criteria defined in proposed compendial methods (Rb2/Rb1 ratio > 0.2)^16^. In addition, the levels of malonyl-ginsenosides were also measured. However, none of the red ginseng samples met the compendial requirement that no peak of malonyl-ginsenoside should be observed (Table 1S, Supporting Information). In terms of detecting the species origin, the current method was also validated using additional lots of market ginseng samples at a different location (Table 2S, Supporting Information).

### Determine the limit of detection of *P. ginseng* tetra-primer ARMS-PCR using mixture

To further determine the limit of detection of the current assay for mixed botanical origin, market samples used in previous validation were mixed to create artificial ginseng material mixture. For DNA-based assay, the most critical factor that influences test sensitivity is DNA template ratio between target species and potential adulterants, which is a function of material mass ratio, sample conditions, and DNA extraction efficiency. To reduce the number of combinations between *P. ginseng* and other ginseng need to be tested, sample conditions and DNA extraction efficiency factors were simplified by using the *P. ginseng* sample with the highest DNA concentration and one of the other species samples that showed lowest DNA concentration in previous DNA extractions. For unprocessed or lightly processed ginseng roots, *P. quinquefolius* adulteration in *P. ginseng* could be detected starting at 5%/95% mass ratio and consistently detected at 10%/90% mass ratio using both capillary electrophoresis (Fig. 5C) and regular agarose gel electrophoresis (Fig. 8S, Supporting Information). The detection of *P. notoginseng, P. japonicus*, and *E. senticosus* adulteration in *P. ginseng* were archived at 50%/50%, 50%/50%, 40%/60% mass ratio, likely due to low DNA extraction efficiency in other ginseng roots (Fig. 9S, Supporting Information). The detection limit was further improved to 5%/95% mass ratio for all decoctions made from mixed materials (Fig. 5D).

## Discussion

### Tetra-primer ARMS-PCR provides internal control to facilitate result interpretation

Appropriate internal controls are often required to assess PCR inhibitors and DNA degradation, which are the two major impediments to the successful amplification of plant DNA^17,18^. The current tetra-primer ARMS-PCR method offers a direct assessment of both PCR inhibitor and DNA degradation. The successful amplification of the control fragment by the outer primer pair indicates not only the target region is amplifiable, but also the template required for diagnostic fragment amplification is intact. Therefore, the absence of the small diagnostic fragment could be confidently interpreted as a true negative.

### Oligo modifications to improve assay sensitivity and specificity

The Taq DNA polymerase is essential to ARMS-PCR due to its lack of 3′ to 5′ exonuclease activity, so it cannot excise mismatched bases like other proofreading polymerases^19^. However, Taq DNA polymerase also possesses 5′ to 3′ exonuclease activities (TaqMan activity), which can hydrolyze the downstream non-template inner fragment primer and its daughter strand^20^. In the current assay, theoretically, the inner primer could bind to two types of templates and lead to different fates, as illustrated in Fig. 6A. When the inner primer binds to a longer template that also contains complement sequence for the outer primer, it has a high chance of being hydrolyzed by upstream Taq DNA polymerase while extending from the outer primer, resulting in weaker intensity of the diagnostic bands. In contrast, the inner primer and its daughter strand will not be hydrolyzed when it binds to the shorter template (synthesized in the amplification process) that has no upstream binding site for the outer primer. To reduce Taq DNA polymerase′s undesired activity on bound inner primer, various strategies have been tested^21^. Among these strategies, using Q5 DNA polymerase achieves the best result. However, its 3′ to 5′ exonuclease activity prevents it from being used in ARMS-PCR. In the current study, a novel strategy that utilizes an inner primer with a 5′ terminus random nucleotide, which contains roughly 75% of bases that are not the wild-type bases, showed success. When these primers are extended by Taq DNA polymerase, a similar portion of artificial short templates (that start with C, G, and T, but not A) are created. As a result, inner primers with a 5′ mismatch to the wild-type sequence shall have a better hybridization affinity to these artificial short templates than its competitor, the long wild-type sequence. In each PCR cycle, a slightly higher percentage of inner primers will be preserved to improve the sensitivity of target species detection (Fig. 6B). Our results showed both random nucleotides (Fig. 3) and random nucleotides other than the wild-type sequence (Fig. 1S, Lane 2 and 6, Supporting Information) on the 5′ terminus of inner primer have similar benefits. However, simply adding random nucleotides on the 5′ terminus of inner primer would be an straightforward solution.

The specificity of tetra-primer ARMS-PCR mainly depends on the reduced elongation efficiency of Taq DNA polymerase at the template-primer 3′ terminus with mismatched base pairs^22^. Ye et al. suggested that an additional deliberate mismatch at the − 2 position from 3′ terminus could further improve specificity^11^. In the current study, the same strategy was tested, and an alternative solution was proposed to further improve assay specificity. By replacing regular phosphodiester bonds at the 3′ terminus with phosphorothioate linkages, it not only makes the oligo more resistant to nuclease, but also renders the oligo more rigid, therefore improving assay specificity in high PCR cycles^23^. Combining the modifications on both the 5′ and 3′ ends, the current assay is suitable to test both raw and lightly processed ginseng material under one set of conditions. This modification strategy has universal application, since similar improvement effects were observed in other species identifications with different primer sequences (Fig. 10S, Supporting Information).

### Simultaneous species confirmation and adulteration detection by tetra-primer ARMS-PCR

In commercial trade, adulteration that involves complete replacement of *P. ginseng* material with material of another species is rare. However, partially substitution of *P. ginseng* material with another species occurs more frequently. The mixed material presents challenges for both chemical and genomic identification methods used in daily quality control practice. In the current study, several lots of red ginseng slices with mixed botanical origin were still able to pass the ginsenoside Rb2/Rb1 ratios acceptance criteria for *P. ginseng* root and red ginseng, even though their ratio is rarely above threshold. On the other hand, genomic methods such as singleplex species-specific PCR, are useful for identification due to the presence of *P. ginseng* DNA in a mixture and the specificity of PCR. However, to provide a fit-for-purpose assay, species-specific PCR for both the target species and its adulterants needs to be used in combination, which increases task complexity in routine testing. The tetra-primer ARMS-PCR assay is designed, based on a multiplex PCR system, which yields specific amplification products for both target species and its adulterants. Our data demonstrates the ability of the assay to give accurate results that match sample species identity with greater sensitivity than traditional HPTLC and HPLC methods for detecting adulteration of *P. ginseng* with other species, in mixed states. In addition, compared to traditional chemical methods, genomic methods generate little chemical waste and can be easily automated for high throughput analysis. A summary of these performance features compared among current methods and other available methods can be found in Supporting Information Table 3S.

## Conclusion

This modified tetra-primer ARMS-PCR method accomplishes target species confirmation and adulteration detection in one test and provides an attractive solution for authentication of *P. ginseng* products′ botanical origin. The application of the same concept can also be extended to the authentication of species identity in other botanical products.

### Supplementary Information

Supplementary Information.

## Data Availability

The dataset(s) supporting the conclusions of this article is(are) included within the article (and its additional file(s).

## Abbreviations

ARMS-PCR: Amplification refractory mutation system
bp: Base pairs
BRM: Botanical reference materials
BLAST: Basic local alignment search tool
HPTLC: High-performance thin-layer chromatography
HPLC: High-performance liquid chromatography
SNP: Single-nucleotide polymorphism

## References

[1] Baeg I-H, So S-H (2013). The world ginseng market and the ginseng (Korea). J. Ginseng Res..

[2] FutureMarketInsights. *Global Ginseng Market: Forecast, Trend, Analysis & Competition Track - Global Review 2019 to 2027*, <https://www.futuremarketinsights.com/reports/ginseng-market> (2019).

[3] Commission, C. P. & THE, S. P. C. O. F. *Pharmacopoeia of the People′s Republic of China*. (Chemical Industry Press, 2011).

[4] Convention, U. S. P. *The United States Pharmacopeia: The National Formulary*. (United States Pharmacopeial Convention, 2013).

[5] Food, K. & administration, d. *Korean Herbal Pharmacopoeia*. (Korea food and drug administration, 2003).

[6] Pawar RS, Handy SM, Cheng R, Shyong N, Grundel E (2017). Assessment of the authenticity of herbal dietary supplements: Comparison of chemical and DNA barcoding methods. Planta Med..

[7] Gafner S (2023). Botanical ingredient forensics: Detection of attempts to deceive commonly used analytical methods for authenticating herbal dietary and food ingredients and supplements. J. Nat. Prod..

[8] Lo Y-T, Li M, Shaw P-C (2015). Identification of constituent herbs in ginseng decoctions by DNA markers. Chin. Med..

[9] Lu Z (2021). Development and validation of a species-specific PCR method for the identification of ginseng species using orthogonal approaches. Planta Med..

[10] Newton C (1989). Analysis of any point mutation in DNA. The amplification refractory mutation system (ARMS). Nucleic Acids Res..

[11] Ye S, Dhillon S, Ke X, Collins AR, Day IN (2001). An efficient procedure for genotyping single nucleotide polymorphisms. Nucleic Acids Res..

[12] Katoh K, Rozewicki J, Yamada KD (2019). MAFFT online service: Multiple sequence alignment, interactive sequence choice and visualization. Brief. Bioinform..

[13] Team, R. C. R: A language and environment for statistical computing. (2013).

[14] Wickham H (2011). ggplot2. Wiley Interdiscip. Rev. Comput. Stat..

[15] Little S (2001). Amplification-refractory mutation system (ARMS) analysis of point mutations. Current Protocols Hum. Genetics.

[16] Pharmacopeia, U.S. In *United States Pharmacopeia—Pharmacopeial Forum* vol. 46, no 4 (2020).

[17] Porebski S, Bailey LG, Baum BR (1997). Modification of a CTAB DNA extraction protocol for plants containing high polysaccharide and polyphenol components. Plant Mol. Biol. Rep..

[18] Ruhsam M, Hollingsworth PM (2018). Authentication of Eleutherococcus and Rhodiola herbal supplement products in the United Kingdom. J. Pharm. Biomed. Anal..

[19] Eom SH, Wang J, Steitz TA (1996). Structure of Taq polymerase with DNA at the polymerase active site. Nature.

[20] Li Y, Mitaxov V, Waksman G (1999). Structure-based design of Taq DNA polymerases with improved properties of dideoxynucleotide incorporation. Proc. Natl. Acad. Sci. U.S.A..

[21] Shatleh-Rantisi D, Tamimi A, Ashhab Y (2020). Improving sensitivity of single tube nested PCR to detect fastidious microorganisms. Heliyon.

[22] Huang MM, Arnheim N, Goodman MF (1992). Extension of base mispairs by Taq DNA polymerase: Implications for single nucleotide discrimination in PCR. Nucleic Acids Res..

[23] Heissl A, Arbeithuber B, Tiemann-Boege I (2017). High-throughput genotyping with TaqMan allelic discrimination and allele-specific genotyping assays. Methods Mol. Biol..

