# Supplementary material for: Application of a modified tetra-primer ARMS–PCR assay for rapid Panax species identity authentication in ginseng products
Source: Sci Rep. 2023 Sep 1;13:14396. doi: 10.1038/s41598-023-39940-7 (PMC10474259; doi:10.1038/s41598-023-39940-7)
Supplement: Supplementary file 1 — Supplementary Information. [file 41598_2023_39940_MOESM1_ESM.docx]

**Supplementary material**

**Application of a Modified Tetra-primer ARMS–PCR Assay for Rapid** ***Panax ginseng* Species Identity Authentication in Ginseng Products**

Zhengxiu Yang^1¶^, Yat Tung Lo^2^*^¶^, Zheng Quan^3^, Junchen He^1^, Yanjun Chen^1^, Adam Faller^3^, Tiffany Chua^4^, Hoi Yan Wu^2^, Yanjun Zhang^4^, Qiang Zou^1^, Fan Li^1^, Peter Chang^4^, Gary Swanson^4^, Pang Chui Shaw^2*^, Zhengfei Lu^3*^

**Affiliation**

1. Herbalife NatSource (Hunan) Natural Products Co., Quality Control Laboratory, Changsha, 410100, China
2. Li Dak Sum Yip Yio Chin R & D Centre for Chinese Medicine, State Key Laboratory of Research on Bioactivities and Clinical Applications of Medicinal Plants (CUHK) and School of Life Sciences, The Chinese University of Hong Kong, Shatin, N.T., Hong Kong, China
3. Herbalife International of America, Inc., Corporate Center of Excellence Quality Laboratory, 950 W 190th Street, Torrance, CA 90502, USA.
4. Herbalife International of America, Inc., Corporate Quality, 990 W 190th Street, Torrance, CA 90502, USA

¶Contribute equally

*Corresponding

Email: Zhengfei Lu [zhengfeil@herbalife.com](mailto:zhengfeil@herbalife.com); Pang Chui Shaw [pcshaw@cuhk.edu.hk](mailto:pcshaw@cuhk.edu.hk)


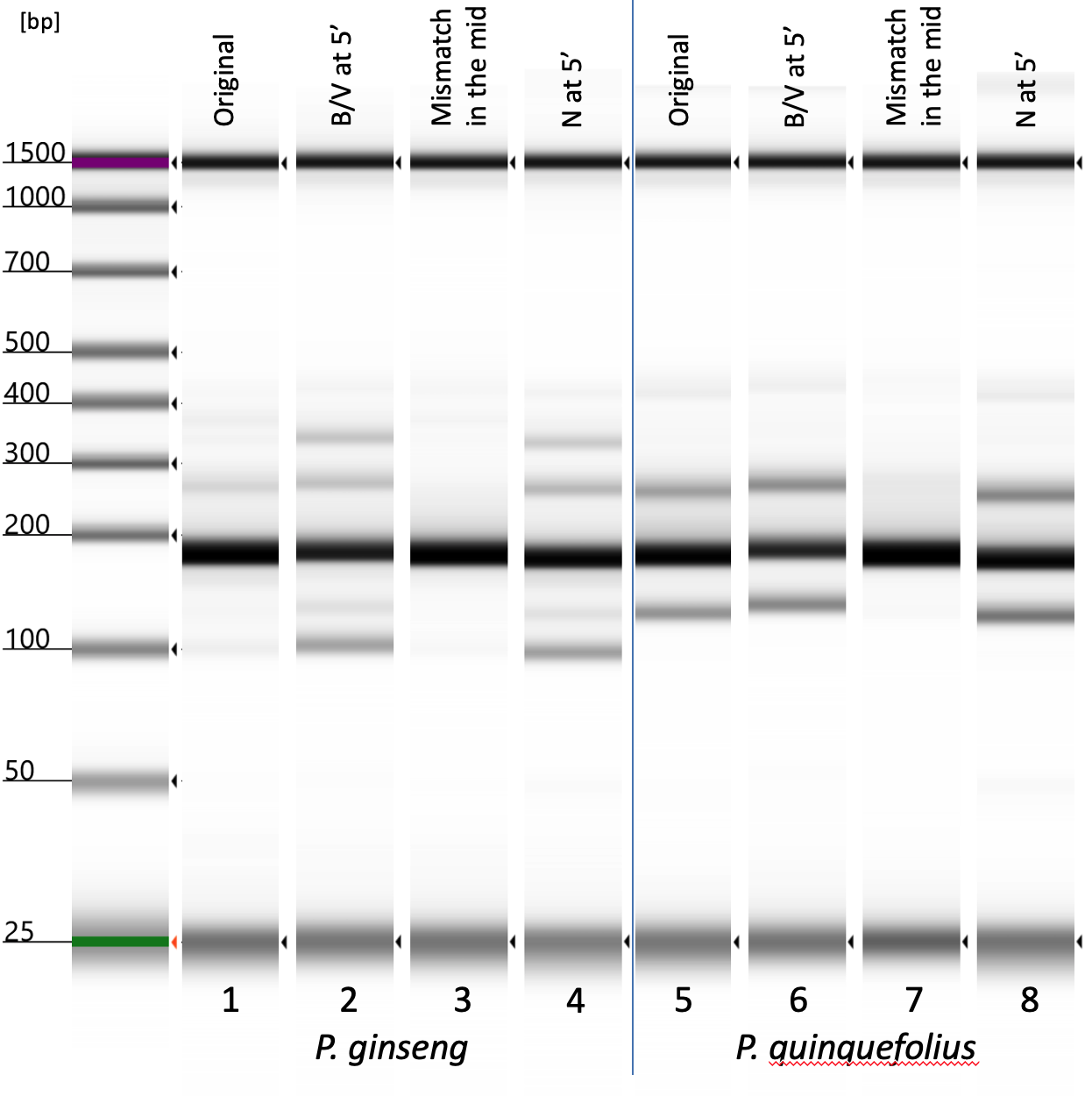


| Lane | Name | Sequence |
| --- | --- | --- |
| 1/5 | Inner F1 | GTCAATACCGGCAACAATGAAATTTT |
|  | Inner R1 | GTCGACGGATTTTCCTCTTACTAT |
| 2/6 | Inner F8 | **V**GTCAATACCGGCAACAATGAAATTTT |
|  | Inner R8 | **B**GTCGACGGATTTTCCTCTTACTAT |
| 3/7 | Inner F9 | GTCAATA**D**CGGCAACAATGAAATTTT |
|  | Inner R9 | GTCGACG**H**ATTTTCCTCTTACTAT |
| 4/8 | Inner F3 | NGTCAATACCGGCAACAATGAAATTTT |
|  | Inner R3 | NGTCGACGGATTTTCCTCTTACTAT |

Fig. 1S. Amplification results from *P. ginseng* and *P. quinquefolius* botanical reference materials using equal ratio of different inner and outer primers at 40 PCR cycles. V is mix of A, G, and C; B is mix of G, C, and T; D is mix of A, G, and T; H is mix of A, C, and T.


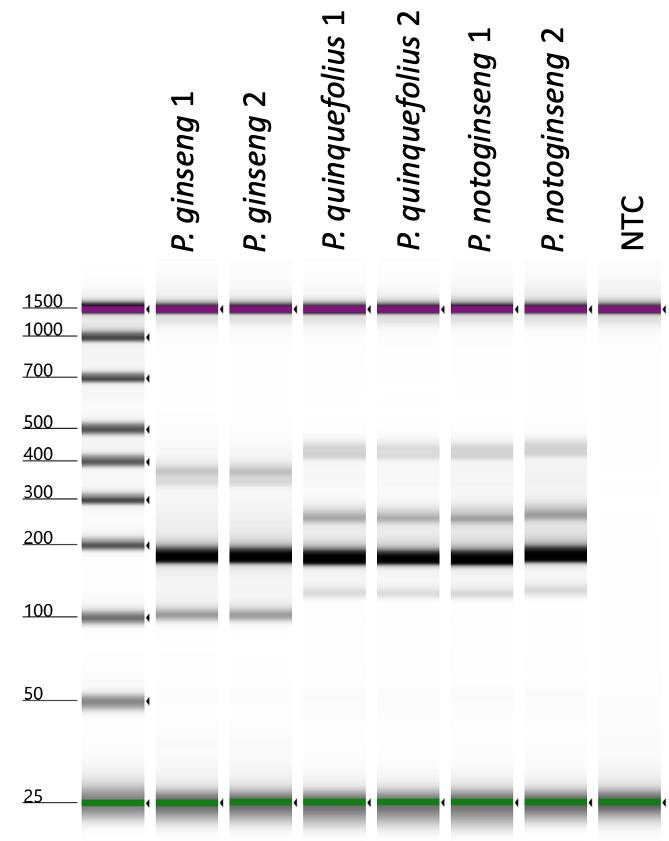


| Name | Sequence | Molar Ratio |
| --- | --- | --- |
| Outer F | TCACCCCATACATAGTCTGATAGTTC | 1 |
| Outer R | GAGTCAAATGGGCTTTTTGG | 1 |
| Inner F7 | NGTCAATACCGGCAACAATGAAA*T*T*T*T | 1 |
| Inner R7 | NGTCGACGGATTTTCCTCTTA*C*T*A*T | 1 |

Fig. 2S. Amplification results from ginseng botanical reference materials using tetra-primer ARMS-PCR contains inner primer with both 5’ terminus random nucleotides and 3’ terminus nucleotide modification at 40 PCR cycles. NTC: non-template control.


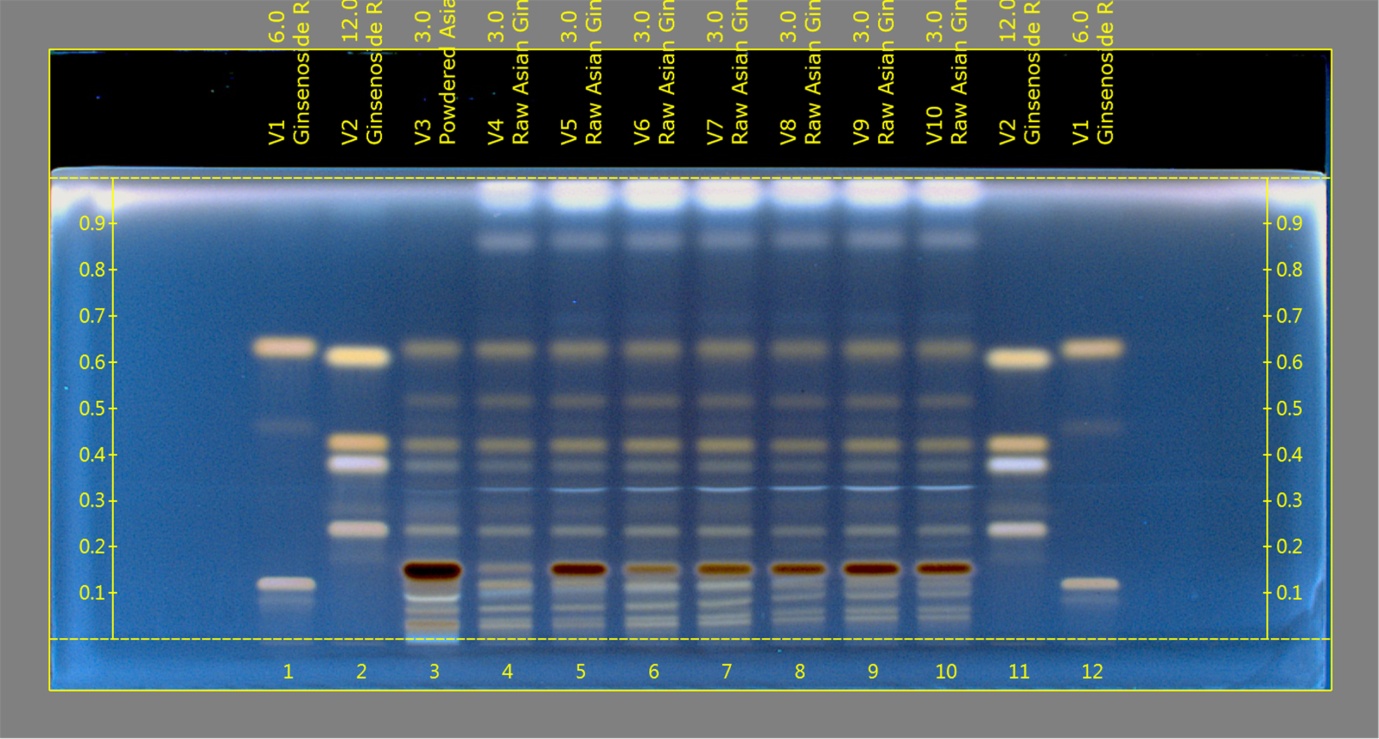

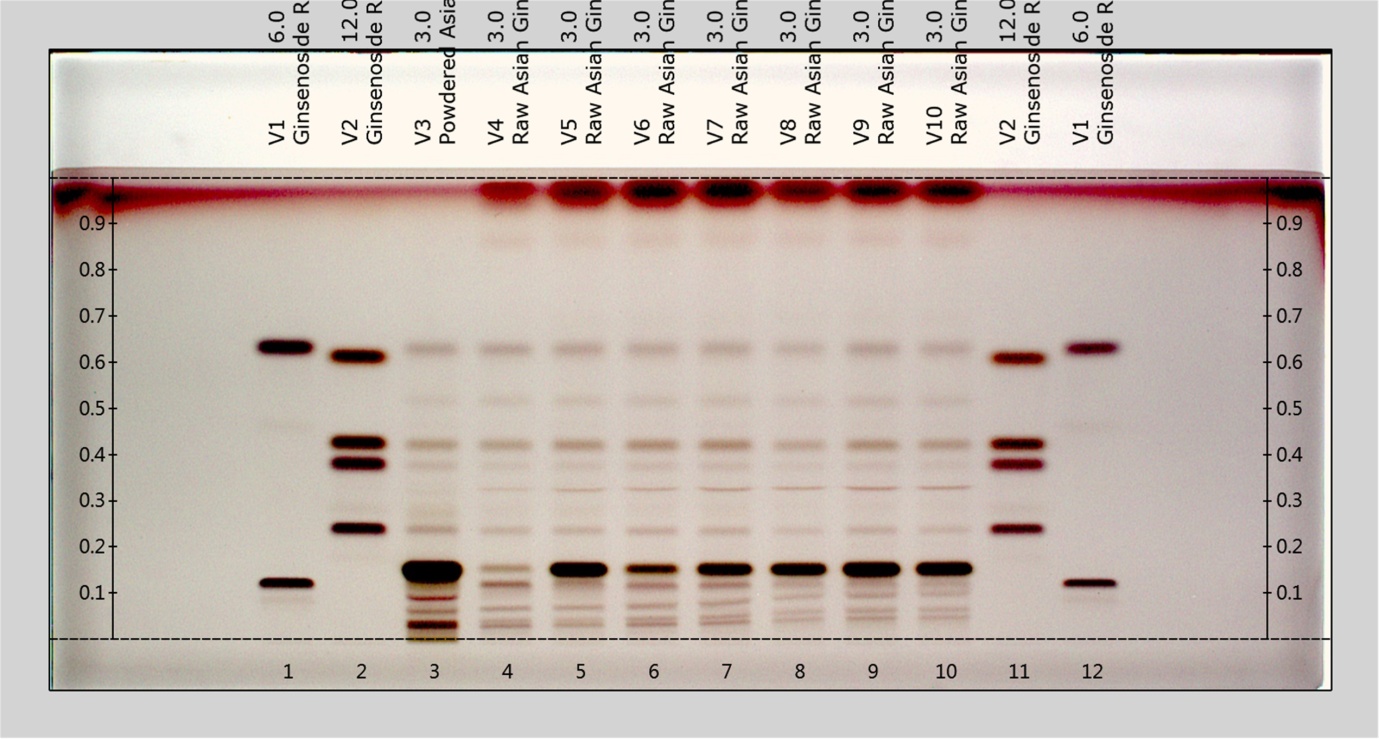


| Track | Sample Name | Volume (µL) |
| --- | --- | --- |
| 1 | Ginsenoside Rb1, Rg1 (increasing Rf) | 6.0 |
| 2 | Ginsenoside Rc,Rd,Re, Pseudoginsenoside F11 (increasing Rf) | 12.0 |
| 3 | Powdered Asian Ginseng Extract RS | 2.0 |
| 4 | Raw Asian Ginseng Root BRM-NIFDC | 2.0 |
| 5 | Raw Asian Ginseng Root-1 | 2.0 |
| 6 | Raw Asian Ginseng Root-2 | 2.0 |
| 7 | Raw Asian Ginseng Root-3 | 2.0 |
| 8 | Raw Asian Ginseng Root-4 | 2.0 |
| 9 | Raw Asian Ginseng Root-5 | 2.0 |
| 10 | Raw Asian Ginseng Root-6 | 2.0 |
| 11 | Ginsenoside Rc,Rd,Re, Pseudoginsenoside F11 (increasing Rf) | 12.0 |
| 12 | Ginsenoside Rb1, Rg1 (increasing Rf) | 6.0 |

Fig. 3S. HPTLC chromatograms of *P. ginseng* root and rhizome samples. (Top panel) After treated by derivatization reagent, under UV light at 366 nm. (Bottom panel) After treated by derivatization reagent, under white light.


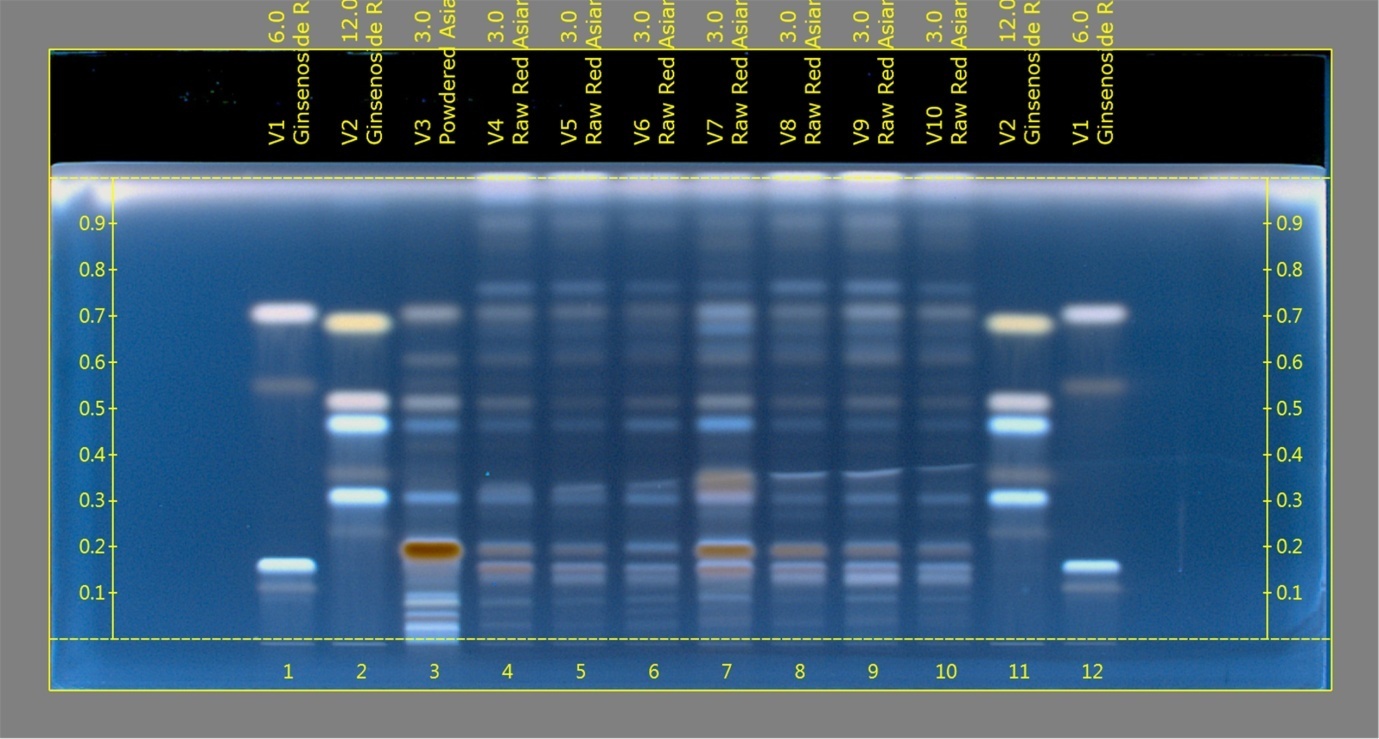

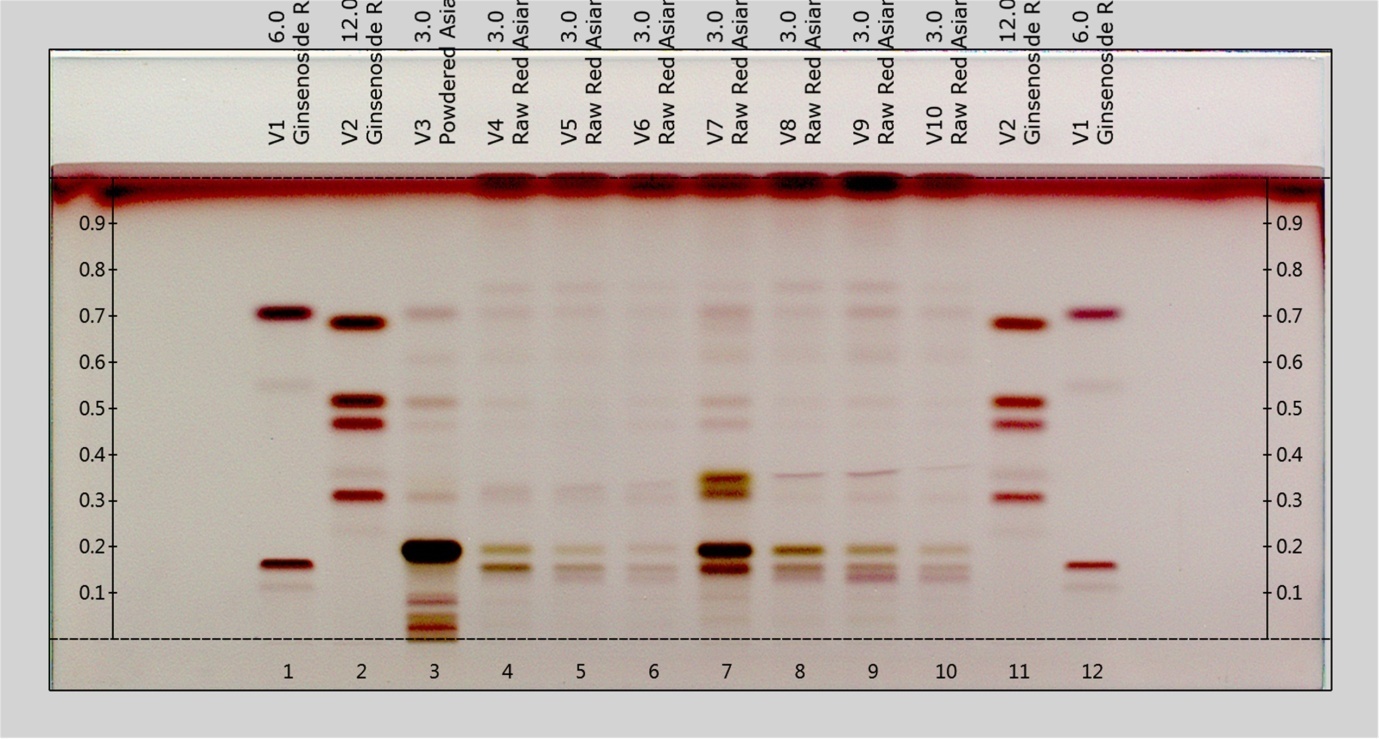


| Track | Sample Name | Volume (µL) |
| --- | --- | --- |
| 1 | Ginsenoside Rb1, Rg1 (increasing Rf) | 6.0 |
| 2 | Ginsenoside Rc,Rd,Re, Pseudoginsenoside F11 (increasing Rf) | 12.0 |
| 3 | Powdered Asian Ginseng Extract RS | 3.0 |
| 4 | Raw Red Asian Ginseng Root BRM-NIFDC | 3.0 |
| 5 | Raw Red Asian Ginseng Root-1 | 3.0 |
| 6 | Raw Red Asian Ginseng Root-2 | 3.0 |
| 7 | Raw Red Asian Ginseng Root-3 | 3.0 |
| 8 | Raw Red Asian Ginseng Root-4 | 3.0 |
| 9 | Raw Red Asian Ginseng Root-5 | 3.0 |
| 10 | Raw Red Asian Ginseng Root-6 | 3.0 |
| 11 | Ginsenoside Rc,Rd,Re, Pseudoginsenoside F11 (increasing Rf) | 12.0 |
| 12 | Ginsenoside Rb1, Rg1 (increasing Rf) | 6.0 |

Fig. 4S. HPTLC chromatograms of red ginseng root and rhizome samples. (Top panel) After treated by derivatization reagent, under UV light at 366 nm. (Bottom panel) After treated by derivatization reagent, under white light.


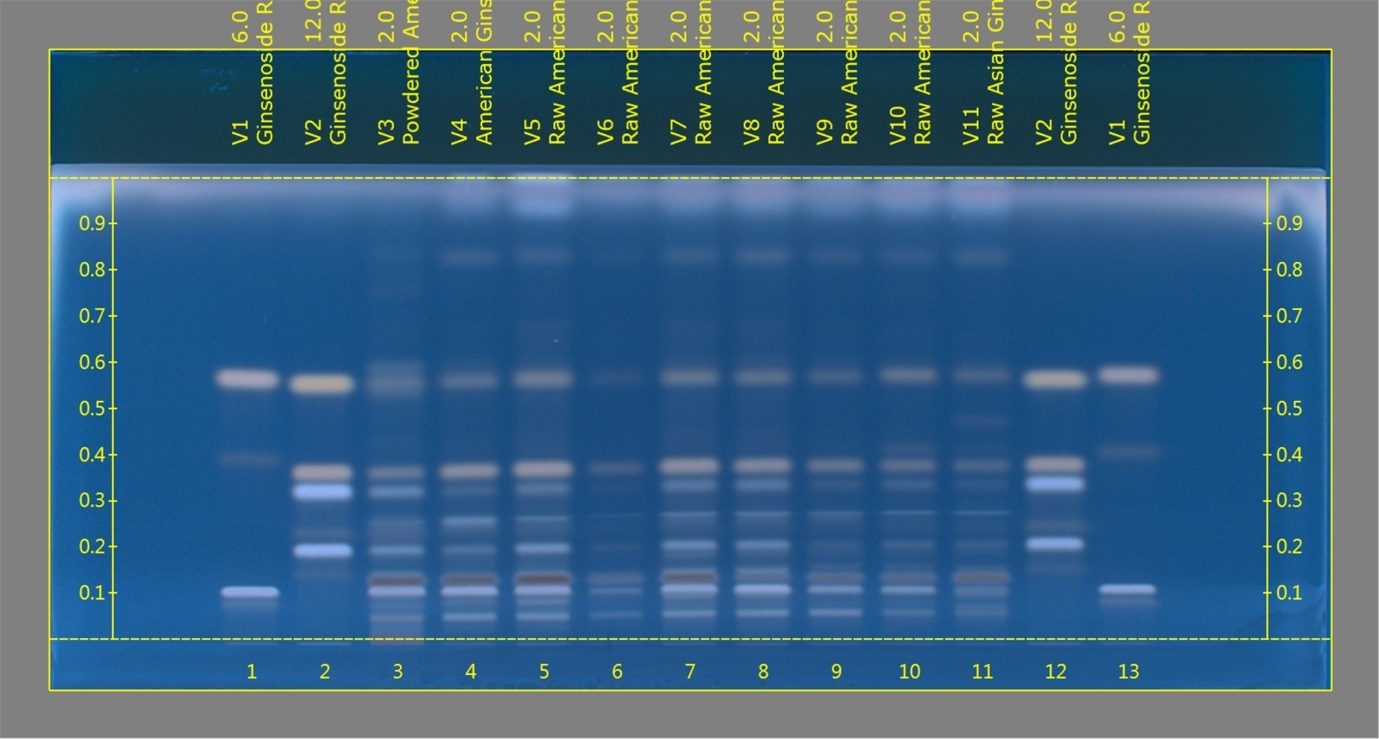

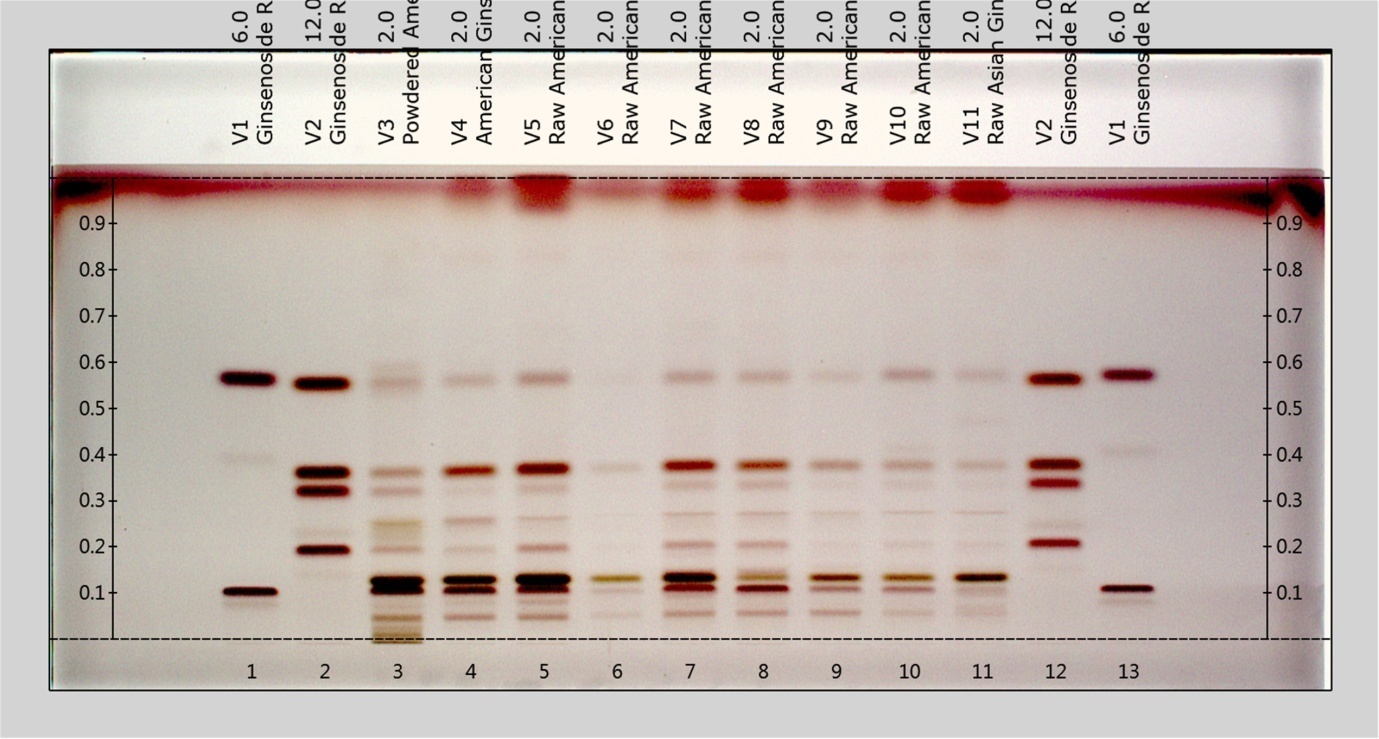


| Track | Sample Name | Volume (µL) |
| --- | --- | --- |
| 1 | Ginsenoside Rb1, Rg1 (increasing Rf) | 6.0 |
| 2 | Ginsenoside Rc,Rd,Re, Pseudoginsenoside F11 (increasing Rf) | 12.0 |
| 3 | Powdered American Ginseng Extract RS | 2.0 |
| 4 | Raw Red Asian Ginseng Root BRM-NIFDC | 2.0 |
| 5 | Raw American Ginseng Root-1 | 2.0 |
| 6 | Raw American Ginseng Root-2 | 2.0 |
| 7 | Raw American Ginseng Root-3 | 2.0 |
| 8 | Raw American Ginseng Root-4 | 2.0 |
| 9 | Raw American Ginseng Root-5 | 2.0 |
| 10 | Raw American Ginseng Root-6 | 2.0 |
| 11 | Raw Asian Ginseng-(+10% American Ginseng) | 2.0 |
| 12 | Ginsenoside Rc,Rd,Re, Pseudoginsenoside F11 (increasing Rf) | 12.0 |
| 13 | Ginsenoside Rb1, Rg1 (increasing Rf) | 6.0 |

Fig. 5S. HPTLC chromatograms of *P. quinquefolius* root and rhizome samples. (Toppanel) After treated by derivatization reagent, under UV light at 366 nm. (Bottom panel) After treated by derivatization reagent, under white light.

HMC_*Panax notoginseng* Root and Rhozome


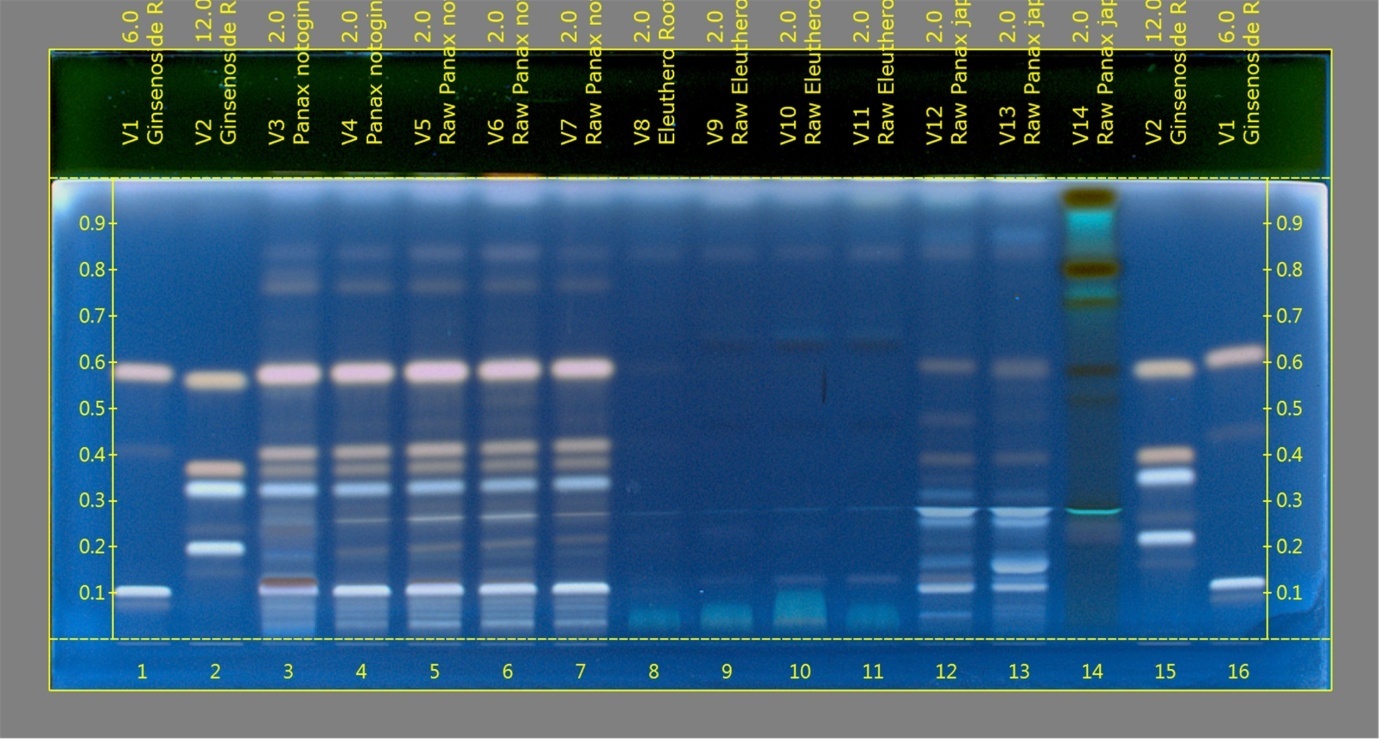


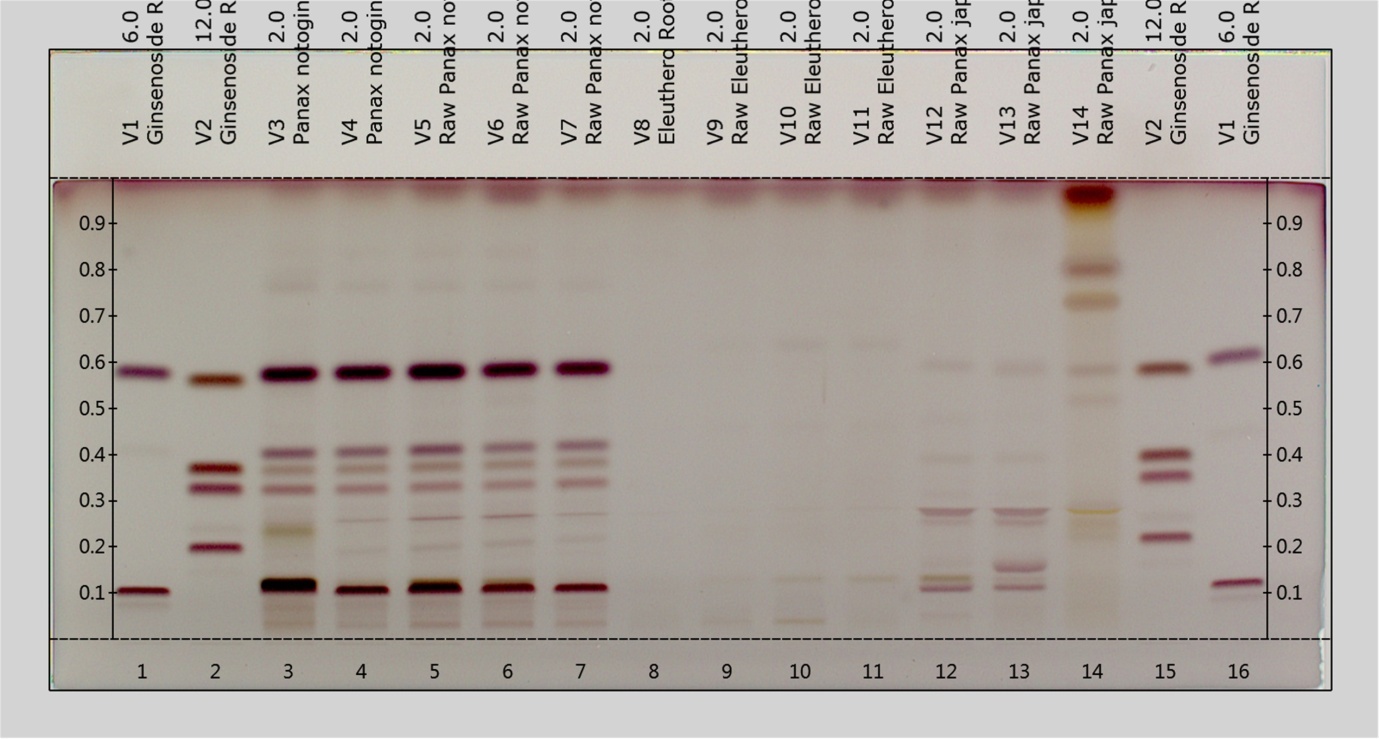


| Track | Sample Name | Volume (µL) |
| --- | --- | --- |
| 1 | Ginsenoside Rb1, Rg1 (increasing Rf) | 6.0 |
| 2 | Ginsenoside Rc,Rd,Re, Pseudoginsenoside F11 (increasing Rf) | 12.0 |
| 3 | *Panax notoginseng* Dry Extract RS | 2.0 |
| 4 | *Panax notoginseng* Root BRM-NIFDC | 2.0 |
| 5 | Raw *Panax notoginseng* Root-1 | 2.0 |
| 6 | Raw *Panax notoginseng* Root-2 | 2.0 |
| 7 | Raw *Panax notoginseng* Root-3 | 2.0 |
| 8 | Eleuthero Root BRM | 2.0 |
| 9 | Raw Eleuthero Root-1 | 2.0 |
| 10 | Raw Eleuthero Root-2 | 2.0 |
| 11 | Raw Eleuthero Root-3 | 2.0 |
| 12 | Raw *Panax japonicus* Root-1 | 2.0 |
| 13 | Raw *Panax japonicus* Root-2 | 2.0 |
| 14 | Raw *Panax japonicus* Root-3 | 2.0 |
| 15 | Ginsenoside Rc,Rd,Re, Pseudoginsenoside F11 (increasing Rf) | 12.0 |
| 16 | Ginsenoside Rb1, Rg1 (increasing Rf) | 6.0 |

Fig. 6S. HPTLC chromatograms of *P. notoginseng, E. senticosus, P. japonicus* root and rhizome samples. (Toppanel) After treated by derivatization reagent, under UV light at 366 nm. (Bottom panel) After treated by derivatization reagent, under white light.


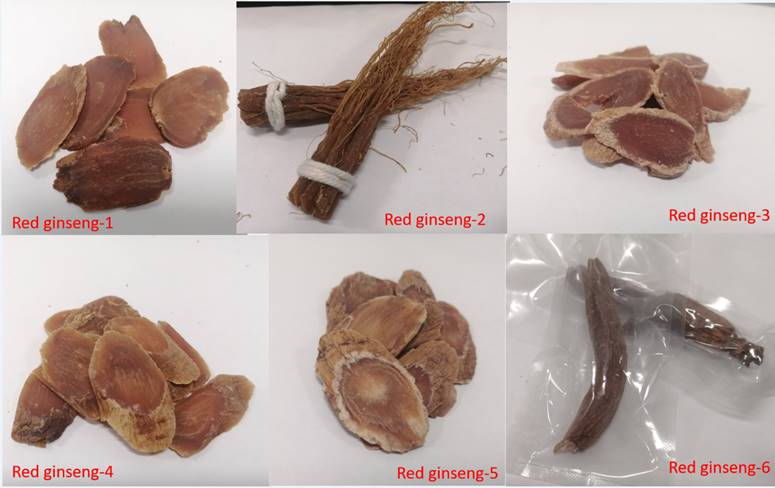


Fig. 7S. Pictures of red ginseng to show sample exist in different forms.


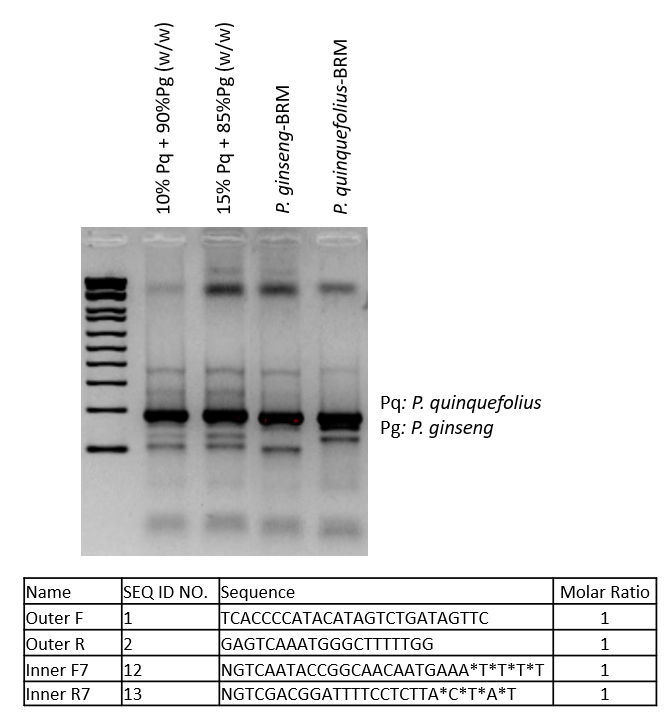


Fig. 8S. Amplification results of *P. ginseng* root mixed with various weight percentage of *P. quinquefolius* root and visualized using agarose gel electrophoresis.


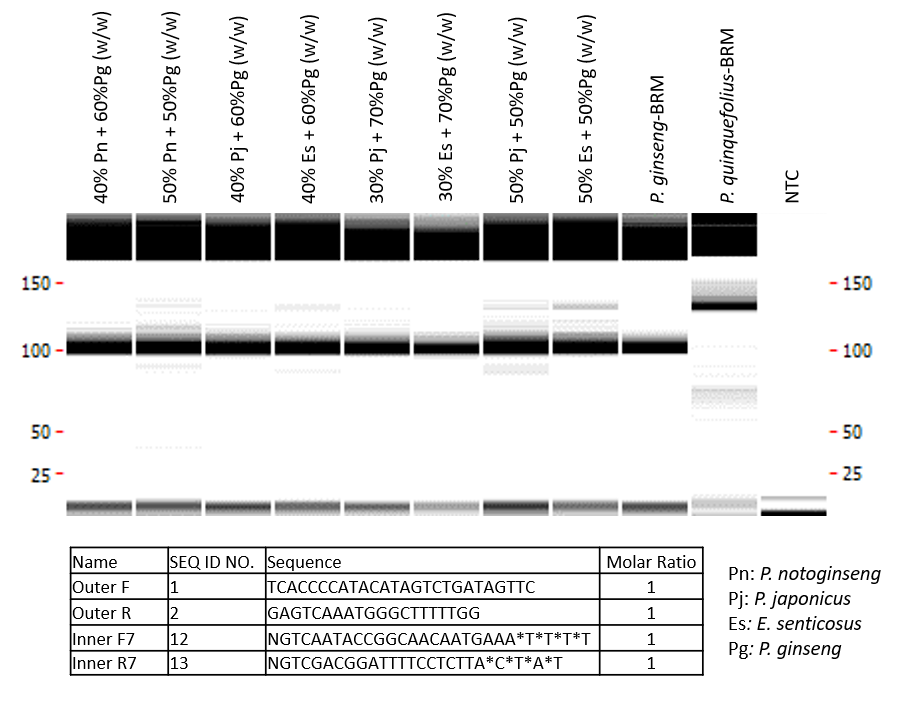


Fig. 9S. Amplification results of *P. ginseng* root mixed with various weight percentage of other species root.


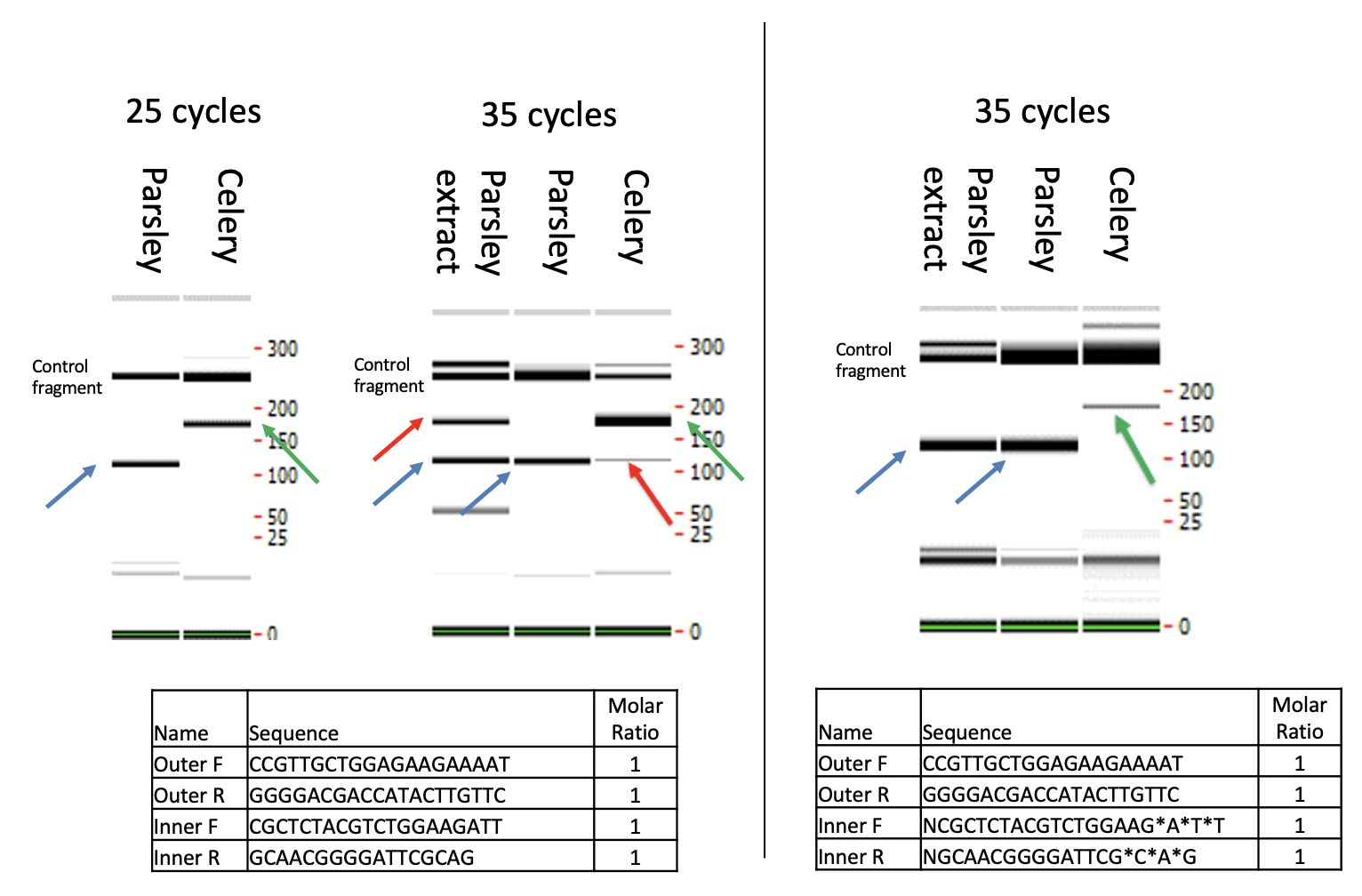


Fig. 10 S. Amplification results of parsley and celery DNA before and after inner primer oligo modification. Blue arrows: parsley specific fragments; green arrows: celery specific fragments; red arrows: non-specific amplification.

| Sample Name | Ginsenoside Peaks (μV*sec) | | | | | | | | | | | Ratios | |
| --- | --- | --- | --- | --- | --- | --- | --- | --- | --- | --- | --- | --- | --- |
|  | Rg1 | Re | Rf | Ro | Rb1 | M-Rb1 | Rc | M-Rc | Rb2 | M-Rb2 | Rd | Rb2/Rb1 | T-M-R/T-R |
| Powdered Asian Ginseng Extract RS | 457118 | 509794 | 146022 | 421276 | 764560 | 365569 | 383222 | 168621 | 352513 | 287604 | 54938 | 0.46 | 0.54775494 |
| American ginseng BRM | 111729 | 955888 | No peak | 283642 | 1219968 | 563394 | 103077 | 127762 | 6890 | 10443 | 37748 | 0.01 | 0.52239795 |
| Red Ginseng BRM | 240198 | 96692 | 67967 | 218025 | 228701 | No peak | 116889 | No peak | 110745 | No peak | 128402 | 0.48 | 0 |
| Panax Ginseng BRM | 391690 | 307891 | 129458 | 189423 | 392870 | 302429 | 200417 | 112025 | 174337 | 207706 | 58949 | 0.44 | 0.81050097 |
| Red ginseng-1 | 130814 | 46999 | 31187 | 91129 | 134000 | 6893 | 48615 | 8767 | 47072 | 3039 | 20209 | 0.35 | 0.08141079 |
| Red ginseng-2 | 110707 | 116465 | 35156 | 98716 | 204439 | 27341 | 162624 | 27230 | 159908 | 26275 | 42155 | 0.78 | 0.15341641 |
| Red ginseng-3 | 61343 | 88160 | 33371 | 77456 | 197429 | No peak | 123597 | 58340 | 116958 | 7404 | 41896 | 0.59 | 0.15010594 |
| Red ginseng-4 | 107872 | 72268 | 27625 | 87534 | 189808 | 16365 | 50093 | 12002 | 41327 | No peak | 32433 | 0.22 | 0.10086833 |
| Red ginseng-5 | 155052 | 76450 | 40324 | 108886 | 143952 | 15780 | 52533 | 20970 | 47600 | No peak | 38824 | 0.33 | 0.1505623 |
| Red ginseng-6 | 151887 | 64960 | 35813 | 107574 | 166243 | 13573 | 64072 | 21337 | 60920 | 5971 | 36645 | 0.37 | 0.14037118 |

Table 1S. Ginsenoside peak sizes in *P. ginseng* extracts, BRM and red ginseng samples. M-Rb1,-Rc, -Rb2: Malonyl-Rb1,-Rc, -Rb2; T-M-R/TR: total content of malonyl ginsenosides to the corresponding neutral ginsenosides.

| Sample code | Item | Authentication results by modified tetra-primer *P. ginseng* ARMS-PCR | |
| --- | --- | --- | --- |
| *Claimed as P. ginseng* | | Raw materials | Decoction |
| T3365 | Whole root raw Asian ginseng | *P. ginseng* | *P. ginseng* |
| T3890 | Asian ginseng | *P. ginseng* | *P. ginseng* |
| T4130 | White ginseng | *P. ginseng* | *P. ginseng* |
| T5254 | Korean ginseng rhizome | *P. ginseng* | *P. ginseng* |
| T5255 | Korean ginseng | *P. ginseng* | *P. ginseng* |
| T5256 | Korean ginseng | *P. ginseng* | *P. ginseng* |
| *Claimed as P. quinquefolius* | | | |
| T5257 | Premium American ginseng slice | Other ginseng | Other ginseng |
| T5258 | American ginseng, size three, two faces | Other ginseng | Other ginseng |
| T5259 | Premium Canadian ginseng slice | Other ginseng | Other ginseng |
| T5260 | American ginseng, jiankou | Other ginseng | Other ginseng |
| T5261 | American ginseng, yuanweixiaozhi | Other ginseng | Other ginseng |
| T2862 | American ginseng cuxu | Other ginseng | Other ginseng |
| *Claimed as P. notoginseng* | | | |
| T2860 | Tienchi | Other ginseng | Not evaluated |
| T2865 | Tienchi | Other ginseng | Not evaluated |
| T3592 | Sanqi | Other ginseng | Not evaluated |
| *Claimed as E. senticosus* | | | |
| T4195 | Siberian ginseng | Other ginseng | Not evaluated |

Table 2S. Market ginseng products or materials used in CUHK laboratory.

| Aspects | Chemical identification methods | | Genomic identification methods | | |
| --- | --- | --- | --- | --- | --- |
|  | HPTLC | HPLC | Barcoding | Species-specific PCR | tetra-primer ARMS-PCR |
| Data interpretation | Subjective interpretation of phytochemical profile and color | Objective | Objective | Objective | Objective |
| Application scope | Material of single origin | Material of single origin | Material of single origin | Material of single origin; must be combined with other PCR methods for mixed sources | Material of single origin and mixed sources with desired sensitivity and specificity |
| Environmental impact | Large amount of solvent usage | Large amount of solvent usage | Little chemical waste generated | Little chemical waste generated | No chemical waste generated |
| Automation feasibility | Manual procedure | Manual procedure | Easy to automate | Easy to automate | Easy to automate |

Table 3S. Comparison between current method and other available methods.

Chemical analysis

HPTLC analysis

HPTLC conditions for authenticating *P. ginseng* roots and *P. quinquefolius* roots were adapted from the USP Asian Ginseng Root and Rhizome, Red Asian Ginseng Root and Rhizome, and American Ginseng Root and Rhizome monograph in *USP-PF* 45(4)^[[1]](#footnote-2)^, which contains the proposed revisions of the USP monographs for Asian Ginseng, Red Asian Ginseng, and American Ginseng [1,2]. HPTLC conditions for *P. notoginseng* were adapted from the Herbal Medicines Compendium *Panax notoginseng* Root and Rhizome (Final Authorized Version 1.0) [3]. *P. japonicus* and *E. senticosus* roots were also evaluated using the same HPTLC condition as *P. notoginseng*. The procedure also involved the performance of the chromatographic resolution under controlled conditions described in the USP General Chapter <203> HPTLC for Articles of Botanical Origin and comparison of the chromatographic profile of the test sample with those of the applicable reference standards (RS). Briefly, ginsenoside Rg1 RS, ginsenoside Rb1 RS, ginsenoside Rc RS, ginsenoside Rd RS, ginsenoside Re RS, pseudoginsenoside F11 RS, powdered Asian ginseng extract RS, powdered American ginseng extract RS, *P. notoginseng* root and rhizome dry extract RS, Asian ginseng root samples, red Asian ginseng root samples, American ginseng root samples and *P. notoginseng* root samples were prepared in alcohol at 0.5 mg/mL (for chemical RS), 10 mg/mL (for extract RS), 200 mg/mL (for Asian and red Asian ginseng root samples), 100 mg/mL (for American Ginseng and *P. notoginseng* root samples) (sonicated and filtered), respectively. The chromatographic system using CAMAG HPTLC system was controlled by visionCATS software, including visualizer, automatic TLC sampler 4, automatic developing chamber, TLC plate heater III, and chromatogram immersion device. Alcohol extracts of finely powdered root sample solutions were applicated 2 µL or 3 µL on silica gel 60 F254, 20 × 10 glass plates as 8-mm bands. A mixture of methylene chloride-anhydrous ethanol-water (60:45:6.5, v/v/v) was used as a developing solvent, condition the plate to a relative humidity of about 33%, temperature of about 25 °C, and the plates were developed to 80 mm distance from the application line in the automatic developing chamber. A solution of 10 % sulfuric acid in alcohol was used as a derivatization reagent, and derivatized plates were heated at 105 °C for 10 min and examined under white RT light and UV light at 366 nm.

UHPLC analysis for red ginseng

UHPLC conditions for the identification of red ginseng roots were adapted from the USP Red Asian Ginseng Root and Rhizome monograph in *USP-PF* 46(4) [4]. The procedure for authenticating red ginseng roots also involved setting up the chromatographic conditions according to the monograph and confirming system suitability for chromatographic resolution under controlled conditions described in the USP General Chapter <621> Chromatography, as well as comparison of the chromatographic profile of the test sample with that of the applicable RS. Specifically, the same set of RS used in HPTLC analysis was used, with variations in concentration and solvent. Briefly, ginsenoside Rg1 RS and ginseng extract RS were dissolved in Methanol-water 7 : 3 (v/v) at 0.15 mg/mL and 10 mg/mL, respectively. For herbal samples, 500 mg of finely powdered root slices were extracted in 25 mL of Methanol-water 7 : 3 (v/v) (30 min sonication and filtration). All ginseng extract RS and sample extracts were passed through a nylon filter of 0.22-µm pore size before injection. The UHPLC system consisted of a Waters Acquity Arc HPLC system coupled to Diode Array Detector (Waters Corp., Milford, MA, USA). A Waters Acquity UPLC BEH C18 1.7µm*2.1mm*50mm, (Part# 186002350) was used for sample analysis. The mobile phase consisted of a combination of A (0.003% of phosphoric acid in water) and B (Acetonitrile). The gradient varied linearly with increasing mobile phase B from 17% to 19% in 3.5 min, from 19% to 23% in 5 min, from 23% to 30% in 15 min, and finally from 30% to 95% in 16.1 min and held at 95% to 20 min, at a flow rate of 0.8 mL/min. The injection volume for standards and samples was 5 µL, and the column temperature was set at 40 °C. The chromatograms were recorded at 203 nm.

1. Pharmacopeia US. Powdered Asian Ginseng. In, United States Pharmacopeia - Pharmacopeial Forum 45 (4); 2019

2. Pharmacopeia US. Powdered American Ginseng. In, United States Pharmacopeia - Pharmacopeial Forum 45 (4); 2019

3. Pharmacopeia US. *Panax notoginseng* Root and Rhizome. In, United States Pharmacopeia - Herbal Medicines Compendium; 2014

4. Pharmacopeia US. Red Asian Ginseng Root and Rhizome. In, United States Pharmacopeia - Pharmacopeial Forum 46 (4); 2020

1. According to USP, documents in USP-PF Online are not official and not suitable to demonstrate compliance. They may never become official. Therefore, proposed revisions and proposed compendial methods are used in this article when referring to those methods. [↑](#footnote-ref-2)
